# Electron-assisted probing of polaritonic light–matter states

**DOI:** 10.1515/nanoph-2023-0907

**Published:** 2024-02-27

**Authors:** Jaime Abad-Arredondo, Antonio I. Fernández-Domínguez

**Affiliations:** Departamento de Física Teórica de la Materia Condensada and Condensed Matter Physics Center (IFIMAC), 16722 Universidad Autónoma de Madrid, E-28049 Madrid, Spain

**Keywords:** quantum emitter, cavity mode, polaritonic state, modulated electron beam, spectroscopy, microscopy

## Abstract

Thanks to their exceptional spatial, spectral and temporal resolution, highly-coherent free-electron beams have emerged as powerful probes for material excitations, enabling their characterization even in the quantum regime. Here, we investigate strong light–matter coupling through monochromatic and modulated electron wavepackets. In particular, we consider an archetypal target, comprising a nanophotonic cavity next to a single two-level emitter. We propose a model Hamiltonian describing the coherent interaction between the passing electron beam and the hybrid photonic–excitonic target, which is constructed using macroscopic quantum electrodynamics and fully parameterized in terms of the electromagnetic dyadic Green’s function. Using this framework, we first describe electron-energy-loss and cathodoluminescence spectroscopies, and photon-induced near-field electron emission microscopy. Finally, we show the power of modulated electrons beams as quantum tools for the manipulation of polaritonic targets presenting a complex energy landscape of excitations.

## 1 Introduction

Much research attention has focused lately on the strong-coupling (SC) phenomena that emerge when quantum emitters (QEs), such as organic molecules, solid-state vacancies, or quantum dots, are placed within the near-field of photonic resonators, such as Fabry–Perot cavities, metamaterial devices, or nanoantennas [1], [2], [3]. In setups involving macroscopic ensembles of QEs, the formation of polaritons (hybrid light–matter states) has opened the way for the manipulation of matter for purposes such as the modification of material properties or the control of chemical reactions [4], [5]. The high complexity of these systems, however, makes their theoretical description extremely challenging, which severely limits the capability of current theories to reproduce experimental results [3], [6]. Complementarily, polariton formation in systems comprising a single (or few) QEs [7], [8], [9] have been investigated for quantum light generation [10], [11] in studies that have also shed light into different aspects of light–matter SC at the macroscopic scale [12]. However, the inherent dark character of these microscopic systems [13], which must feature large light–matter interaction strengths and small radiative losses, prevents their full characterization by far-field, optical means.

Traditional electron-beam-based optical characterization methods [14], [15], such as electron-energy-loss spectroscopy (EELS) or cathodoluminiscence (CL) microscopy, present extraordinary spatial and spectral resolutions, approaching the subnanometric and milielectronvolt ranges, respectively [16], [17]. These make them ideal for the exploration of light–matter SC and polaritonic states in nanophotonic samples involving only a few excitons [18], [19], [20], [21]. Moreover, in the last years, advances in ultrafast optical control of free-electron wavepackets reached the femtosecond scale, matching the optical period of visible light [22]. These are behind the emergence of techniques such as photon induced near-field electron microscopy (PINEM), that exploits the synchronous interaction between free-electrons and spatially-confined pulsed laser fields [23]. Developments in PINEM theory [24], [25] and, generally, in the description of electron–photon interactions [26], [27], [28], together with the extraordinary degree of optical modulation (in time and momentum space) of electron beams attainable today [29], [30], [31], have made possible their use to imprint, exchange and manipulate quantum coherence in optical and material excitations, sustained by micro- and nano-cavities [32], [33], [34], [35], [36], [37] and QEs [38], [39], [40], respectively. Only very recently, similar ideas have been proposed for hybdrid excitonic-photonic systems, where light–matter SC takes place. By means of phenomenological studies, it has been shown theoretically that electron–polariton interactions offer opportunities in areas such as sensing [41] and quantum information [42].

Here, we present a model Hamiltonian describing the quantum interaction between a modulated electron wavepacket and a polaritonic target comprising a single QE (treated as a two-level system) and a nanophotonic cavity. The Hamiltonian is constructed using the framework of macroscopic quantum electrodynamics (QED) [43], [44], [45], [46] and is fully parameterized in terms of the electromagnetic dyadic Green’s function. For simplicity, we consider a cavity with spherical symmetry, and to unveil clearly quantum-coherent effects in the light–matter SC, we restrict its Hilbert space to the lowest (degenerate), dipolar modes that it supports. We explore the polariton energy ladder of the hybrid photonic–excitonic system through both the free-electron wavepacket and photon spectra in EELS-, CL- and PINEM-like setups. Finally, we demonstrate the power of modulated electron beams to probe and control light–matter states in the SC regime.

## 2 Target-probe system and model Hamiltonian

**Figure 1:**
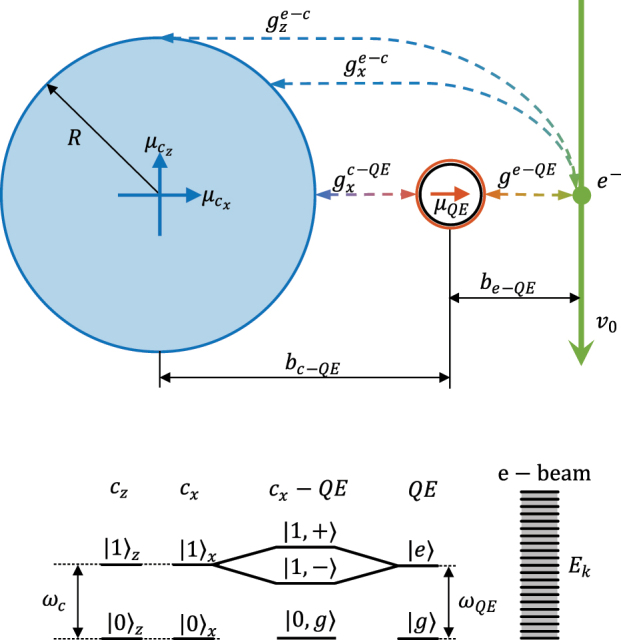
Top: sketch of the system under consideration: an electron wavepacket with central velocity $v_{0}\hat{z}$ and kinetic energy *E*_*k*_ passes through a target system composed of a nanoparicle cavity and a QE. The nanoparticle radius is 10 nm and its spectrum is restricted to two degenerate dipolar cavity modes with energy *ℏω*_*c*_ = *ℏω*_QE_ = 2 eV, at resonance with the QE. Bottom: illustration of the energy levels of $\hat{H_{0}}$ for target (left) and electron beam (right). The *z*-dipolar cavity mode is uncoupled from the QE, while the *x*-dipolar one is strongly coupled to it, giving rise to non-degenerate polaritonic states.

The target-probe system that we have chosen to assess the ability of free electrons to explore light–matter SC is depicted in the top panel of Figure 1. We consider a nanophotonic cavity (typically a metal nanoparticle), sustaining a dipolar-like confined mode overlapping with the dipole moment, *μ*_QE_ = 1 e nm (parallel to *x*-axis), of a QE placed in close proximity of the nanoparticle surface (the QE-cavity distance is similar to the cavity radius itself, *b*_*c*−QE_ ≈ *R*), also along the *x*-direction. The free-electron wavepacket, with energies in the order of 10 keV, passes through the compound target along the $\hat{z}$ direction with impact parameters *b*_*e*−*c*_ and *b*_*e*−QE_ with respect to cavity and QE, respectively. QE and cavity are, unless specified otherwise, at resonance, with *ℏω*_*c*_ = *ℏω*_QE_ = 2 eV. This enables us to neglect the contribution from higher order, multipolar modes in the QE-cavity interaction. To maximize their coupling, we set *R* = 10 nm, which corresponds to modal dipole moments of $\mu_{c_{x,y}}=40\;\text{e}$ nm [47]. In the following, we employ this idealized, but feasible, system as a test-bed to explore the phenomenology resulting from the electron probing of polaritonic states.

In Sections S1–4 of the Supplementary Material (SM), we provide details of the derivation of the system Hamiltonian and its parametrization using macroscopic QED. The small size of the cavity allows us to use the quasi-static approximation for the dyadic Green’s function employed in the electromagnetic description of the target and passing electrons. The impact of retardation and nonlocal effects, beyond the quasi-static picture, is also discussed in Section S4 of the SM. The system Hamiltonian can be written as $\hat{H}=\hat{H_{0}}+\hat{H_{I}}$ , with

(1) $$\begin{array}{ll} \hat{H_{0}} & =\hbar \underset{i=x,z}{\sum }\omega_{c}{\hat{a}}_{i}^{†}{\hat{a}}_{i}+\hbar \omega_{\text{QE}}{\hat{\sigma }}^{†}\hat{\sigma }+\\ & \;+\underset{k}{\sum }E_{k}{\hat{c}}_{k}^{†}{\hat{c}}_{k}+\hbar g_{x}^{c-\text{QE}}\left[{\hat{a}}_{x}^{†}\hat{\sigma }+{\hat{a}}_{x}{\hat{\sigma }}^{†}\right], \end{array}$$

(2) $$\begin{array}{ll} \hat{H_{I}} & =+\hbar \underset{i=x,z}{\sum }\underset{q}{\sum }\;g_{q,i}^{e-c}{\hat{b}}_{q}\left[{\hat{a}}_{i}^{†}-{\hat{a}}_{i}\right]\text{sign}\left(q\right)\\ & \;+\hbar \underset{q}{\sum }g_{q}^{e-\text{QE}}{\hat{b}}_{q}\left[\hat{\sigma }-{\hat{\sigma }}^{†}\right]\text{sign}\left(q\right). \end{array}$$

$\hat{H_{0}}$ describes the free dynamics of target and electron beam independently, and $\hat{H_{I}}$ their interaction. This Hamiltonian captures the terms previously used to study free electron interaction with optical modes [34], [35], [36], QEs [38], [39], [40], and polaritonic systems [41], [42]. We also note that the parametrization through macroscopic QED allows retrieving the classical results from EELS theory (see Section S4 of the SM). Also importantly, the general form of the Hamiltonian parameters in terms of the dyadic Green’s function provided in Sections S1 and 2 of the SM allows the investigation of electron–polariton interactions beyond our model system, to other experimentally relevant nanophotonic platforms.

In Equations (1) and (2), ${\hat{a}}_{i}$ (*i* = *x*, *z*) are the annihilation operators for the degenerate dipolar cavity modes (note that, by symmetry, we can consider only those within the *xz*-plane in Figure 1), $\hat{\sigma }=\left|g\right\rangle \left\langle e\right|$ is the two-level-system lowering operator for the QE excitons, and *c*_*k*_ is the operator describing the annihilation of free-electron population in the wavepacket component with momentum *k* and energy *E*_*k*_ = (*ℏk*)^2^/2*m*_*e*_. The fourth term in Equation (1) accounts for the cavity-emitter coupling in the rotating wave approximation with strength (see Section S4 of the SM)

(3) $$g_{x}^{c-\text{QE}}=\frac{\omega_{\text{QE}}}{3}\sqrt{\frac{\pi }{2}{\left(\frac{R}{b_{c-\text{QE}}}\right)}^{3}\frac{\mu_{\text{QE}}^{2}}{\hbar \omega_{c}\epsilon_{0}b_{c-\text{QE}}^{3}}}.$$

Note that the QE only couples with the cavity mode with an effective dipole moment along *x*-direction.

The two Holstein-like terms in Equation (2) describe the target-probe interaction, where ${\hat{b}}_{q}=\sum_{k}{\hat{c}}_{k-q}^{†}{\hat{c}}_{k}$ is the ladder operator that shifts the free-electron momentum by an amount *q*, which is transferred to or from the cavity modes (first terms) or QE exciton (last term). Note that, contrary to the QE, the passing electrons couple to both the *x*- and *z*-dipolar cavity modes. As detailed in Sections S3 and 4 of the SM, the electron-cavity and electron-QE coupling strenghts can be written as

(4) $$g_{q,x}^{e-c}=\frac{e\hbar k_{0}}{3m_{e}L}q^{2}K_{1}\left(|q|b_{e-c}\right)\sqrt{\frac{1}{\hbar \epsilon_{0}}\frac{\pi }{2}\frac{R^{3}}{\omega_{c}}},$$

(5) $$g_{q,z}^{e-c}=\frac{e\hbar k_{0}}{3m_{e}L}q^{2}K_{0}\left(|q|b_{e-c}\right)\sqrt{\frac{1}{\hbar \epsilon_{0}}\frac{\pi }{2}\frac{R^{3}}{\omega_{c}}},$$

(6) $$g_{q}^{e-\text{QE}}=\frac{ek_{0}q^{2}\mu_{\text{QE}}}{2\pi m_{e}L\epsilon_{0}\omega_{\text{QE}}}K_{1}\left(|q|b_{e-\text{QE}}\right),$$

where *ℏk*_0_ = *m*_*e*_ *v*_0_ ≫ *ℏ*|*q*| is the incoming momentum of the passing electrons, which is ∼4 orders of magnitude larger than the momentum they exchange with the cavity-emitter target (|*q*|∼ *ω*_*c*,QE_/*v*_0_). This fact enables us to operate under the so-called nonrecoil approximation [14]. *K*_0.1_(⋅) are modified Bessel functions of the second kind, and we have assumed positive impact parameters (*b*_*i*−*j*_ > 0 for all *i*, *j*). *L* is a length scale introduced in the particle-in-a-box quantization of the electron momentum, we anticipate that all the physical observables calculated in the following will not depend on this quantity, formally introduced for clarity.

We are interested in employing the electron beam as a tool to explore light–matter SC in the target. Therefore, we will proceed by diagonalizing (analytically) the bare Hamiltonian, $\hat{H_{0}}$ , accounting for the cavity-QE interactions at all orders in the coupling strength $g_{x}^{c-\text{QE}}$ and obtaining the polaritonic eigenstates of the target. On the contrary, taking advantage of the fact that the incoming electrons only alter the target weakly, the interaction Hamiltonian, $\hat{H_{I}}$ , will be treated perturbately, only considering processes up to second order of interaction in $g_{q,i}^{e-c}$ and $g_{q}^{e-\text{QE}}$ . The bottom panel of Figure 1 illustrates the energy levels of the target (left) and electrons (right). Note that the energy scales are very different as, as indicated above, *E*_*k*_ ≫ *ℏω*_*c*,QE_. The sketch of the ground and first excitation manifolds for the target shows an uncoupled *z*-dipolar cavity mode and the emergence of polaritonic states as a result of the hybridization of the *x*-dipolar cavity mode and the QE exciton. The eigenstates of ${\hat{H}}_{0}$ can be expressed as a product of the free electron states, $\left|k\right\rangle$ , the Fock states of the uncoupled cavity mode, ${\left|n\right\rangle }_{z}$ , and the polaritonic states. If cavity and QE are at resonance (which is the reference configuration for our study), these can be simply written as $\left|N,\pm \right\rangle =\left({\left|N\right\rangle }_{x}\left|g\right\rangle \pm {\left|N-1\right\rangle }_{x}\left|e\right\rangle \right)/\sqrt{2}$ in the *N*th manifold, with energies $\hbar \omega_{N,\pm }=\hbar \left(N\omega_{c,\text{QE}}\pm \sqrt{N}g_{x}^{c-\text{QE}}\right)$ [1], [2], [5]. Therefore, we have

(7) $$\hat{H_{0}}\left|\phi \right\rangle =E_{\phi }\left|\phi \right\rangle ,$$

for the bare system, with $\left|\phi \right\rangle ={\left|n\right\rangle }_{z}⊗\left|N,\pm \right\rangle ⊗\left|k\right\rangle$ and *E*_*ϕ*_ = *ℏω*_*c*_ *n*_*z*_ + *ℏω*_*N*,±_ + *E*_*k*_.

## 3 Electron-target interaction

We use the scattering matrix formalism [37], [38], [39] to describe the alteration of the target states by the passing electrons, which amounts to applying the propagator for the interaction Hamiltonian in the interaction picture $\hat{S}\left(t\right)=Texp\left(-\frac{i}{\hbar }\int_{-\infty }^{t}{\hat{H}}_{I,int}\left(\tau \right)d\tau \right)$ , with T being the time ordering operator, and ${\hat{H}}_{I,int}\left(\tau \right)={\text{e}}^{\text{i}{\hat{H}}_{0}\tau /\hbar }{\hat{H}}_{I}{\text{e}}^{-\text{i}{\hat{H}}_{0}\tau /\hbar }$ . The plasmonic nature of the cavity translates into optical mode lifetimes in the range of several tens of femtoseconds, while the QE lifetime is of the order of hundreds of ps. The electron-target interaction time can be estimated from the ratio *λ*_*c*_/4*v*_0_ ≃ 2 fs (where we have assumed a size for the subwavelengh-confined cavity mode of *λ*_*c*_/4), which is at least one order of magnitude faster than the lifetime of the target states [48]. Thus, using the quasi-instantaneous character of the electron-target interaction, we can describe the mixing of $\left|\phi \right\rangle$ that they induce through

(8) $$\hat{S}\left(t\to \infty \right)=\hat{S}=exp\left(-i\underset{\phi ,\phi^{\prime }}{\sum }h_{I,\phi ,\phi^{\prime }}|\phi 〉\;〈\phi^{\prime }|\right),$$

(9) $$h_{I,\phi ,\phi^{\prime }}=\delta_{E_{\phi },E_{\phi^{\prime }}}\frac{L}{\hbar v_{0}}\left\langle \phi |{\hat{H}}_{I}|\phi^{\prime }\right\rangle .$$

The Kronecker delta in Equation (9) accounts for energy conservation in the electron-target interaction. It is obtained by taking the discrete limit of the continuous delta function [38], $\delta \left(\left(E_{\phi }-E_{\phi^{\prime }}\right)/\hbar \right)\to \left(L/2\pi v_{0}\right)\delta_{E_{\phi },E_{\phi^{\prime }}}$ , which is a consequence of the particle-in-a-box quantization of the electron wavepacket. This introduces a discrete resolution in wave-vector Δ*k* = 2*π*/*L*, and energy Δ*E*_*k*_ = *hv*_0_/*L*. The *L* factor in Equation (9) cancels with the 1/*L* factors in the expectation values $\left\langle \phi \right|{\hat{H}}_{I}\left|\phi^{\prime }\right\rangle$ embeded in the coupling strengths in Equations (4)–(6), which makes the propagator $\hat{S}\left(t\to \infty \right)$ independent of this auxiliary length scale. By relating the initial and final free-electron momenta through the momentum exchanged with the target, *k* = *k*′ − *q*, it is possible to write

(10) $$\delta_{E_{\phi },E_{\phi^{\prime }}}\approx \delta \left(qv_{0},\;\omega_{c}\left(n_{z}-n_{z}^{\prime }\right)+\omega_{N,\pm }-\omega_{N^{\prime },\pm^{\prime }}\right)$$

where we have used the notation *δ*_*i*,*j*_ = *δ*(*i*, *j*) for clarity. Note that in previous works exploring the electron-beam-probing of optical cavities [34] and QEs [38], all the momentum and energy exchanged with the target was in multiples of *ω*_*c*,QE_/*v*_0_ and *ω*_*c*,QE_, since the latter was the only energy scale present in the system. Here, the cavity-QE SC and the resulting polaritonic ladder gives rise to a much more complex landscape of electron-target interactions.

**Figure 2:**
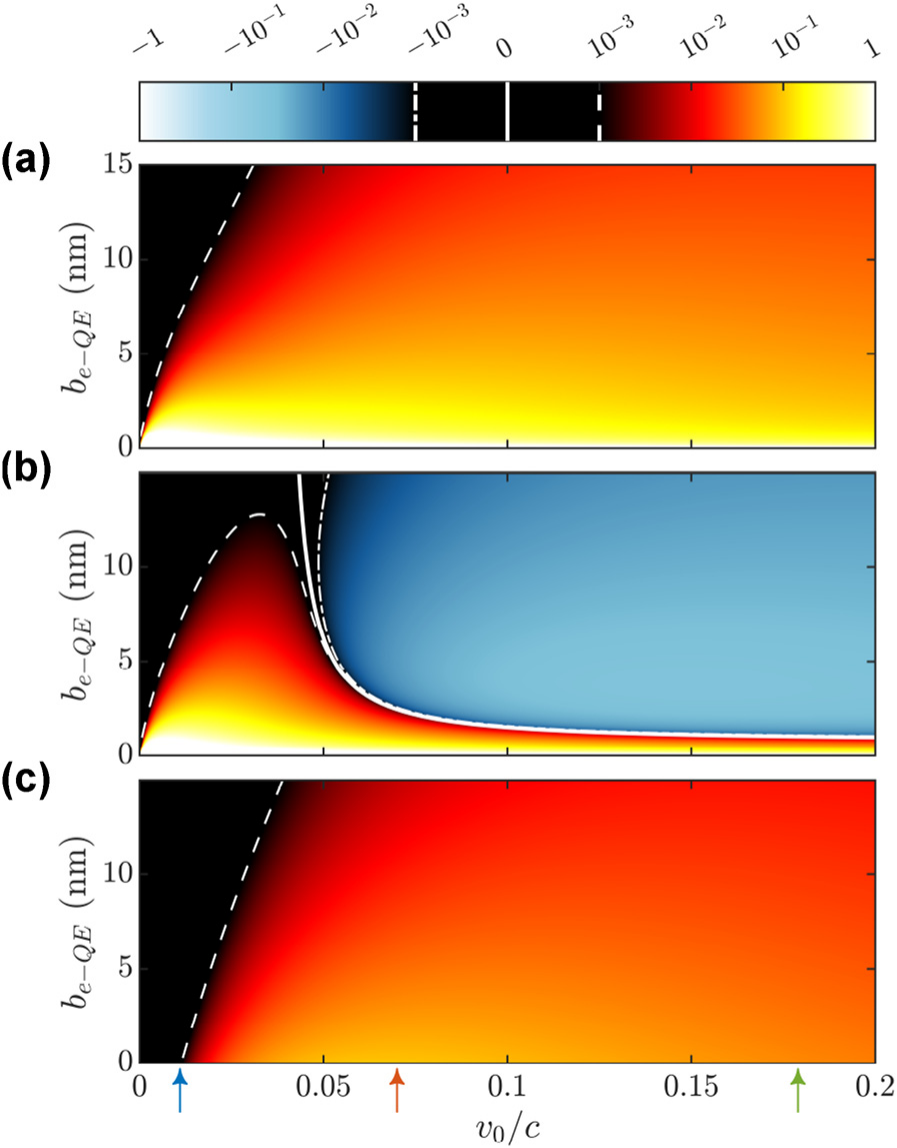
Matrix elements, *h*_*I*,*G*,*ϕ*′_, connecting the target ground state with states of the first excitation manifold as a function of the free electron-QE distance and the electron speed. (a) Upper polariton $\left|1+\right\rangle$ , (b) lower polariton $\left|1-\right\rangle$ , (c) *z* − dipolar mode $\left|1z\right\rangle$ . Note that we are omitting the electronic part of the wavefunction, see main text. Solid, dashed, and dotted-dashed white lines plot the isocurves *h*_*I*,*G*,*ϕ*′_ = 0, *h*_*I*,*G*,*ϕ*′_ = 10^−3^, and *h*_*I*,*G*,*ϕ*′_ = −10^−3^, respectively. The vertical color arrows indicate the configurations considered in Figure 3.

Figure 2 shows the adimensional matrix elements *h*_*I*,*ϕ*,*ϕ*′_ that connect the ground state of the target, $\left|\phi \right\rangle =\left|G\right\rangle ={\left|0\right\rangle }_{z}⊗{\left|0\right\rangle }_{\pm }⊗\left|k\right\rangle$ and the different states of the first excitation manifold of ${\hat{H}}_{0}$ . With the cavity and QE parameters introduced above, we obtain $g_{x}^{c-\text{QE}}\approx 80$ meV, which is in accordance with the light–matter interaction strengths reported experimentally in different nanophotonic-based polaritonic systems at the single QE level [8], [9]. Due to the structure of the interaction Hamiltonian, the evaluation of Equation (9) for $\left|\phi^{\prime }\right\rangle =\left|1\pm \right\rangle ={\left|0\right\rangle }_{z}⊗\left|1,\pm \right\rangle ⊗\left|k-\frac{\omega_{1\pm }}{v_{0}}\right\rangle$ and $\left|\phi^{\prime }\right\rangle =\left|1z\right\rangle ={\left|1\right\rangle }_{z}⊗\left|0\right\rangle ⊗\left|k-\frac{\omega_{c}}{v_{0}}\right\rangle$ yields [41]

(11) $$h_{I,G,1\pm }=\frac{L}{\hbar v_{0}}\left[g_{\omega_{1,\pm }/v_{0},x}^{e-c}\pm g_{\omega_{1,\pm }/v_{0}}^{e-\text{QE}}\right],$$

(12) $$h_{I,G,1z}=\frac{L}{\hbar v_{0}}g_{\omega_{c}/v_{0},z}^{e-c}.$$

Equation (11) illustrates the power of electron beams for the exploration of light–matter SC. In optical-based spectroscopic techniques, which operate under the far-field, laser-like pumping of the polaritonic target, the driving amplitude of the cavity is orders of magnitude larger than the QE. This is a consequence of the dipole mismatch between them, which is $\mu_{c_{x,z}}/\mu_{\text{QE}}≃40$ for the small nanoparticle in our system (see Section S4 of the SM). In these setups, the polariton population takes place through the cavity, and hence, it is exactly the same (except for dispersion effects) for lower and upper states. When employing a very localized excitation, the electron beam in our case, it is possible to make the absolute value of two terms in Equation (11) similar through the tuning of the probe parameters that come into play in the interaction with the target. In this regime, one of the polariton states becomes completely dark to the passing electron, enabling the selective probing of the other one, as all the interaction dynamics will occur solely through it. This phenomenology is similar to the *polariton-blockade* mechanism [42] recently proposed as a resource for quantum information processing.

Figure 2 render *h*_*I*,*G*,1+_ (a), *h*_*I*,*G*,1−_ (b) and *h*_*I*,*G*,1*z*_ (c), as a function of the electron-QE impact parameter, *b*_*e*−QE_, and the central velocity of the electron wavepacket normalized to the speed of light, *v*_0_/*c*. We can observe that all the matrix elements decrease with larger distance and lower velocity (see dashed white lines), although only *h*_*I*,*G*,1−_ completely vanishes within the parameter range considered, as indicated by the white solid line in panel (b). As expected from the setup we have chosen, see Figure 1, the electron probes more efficiently the polaritonic states than the *z*-dipolar cavity mode at small *b*_*e*−QE_. Only at large *v*_0_/*c*, the three panels acquire similar absolute values, although the elements for the lower polariton change sign and become negative in this regime. The study provided in these three panels serves as a guide for the design the electron-beam configuration most appropriate to interrogate a given state of the first excitation manifold in the light–matter SC target.

The adimensional matrix elements in Figure 2 acquire values that range between −1 and 1, which means that the propagator in Equation (8) can be treated perturbatively in different orders of electron-target interaction for most of the configurations analyzed. Note that all the results that follow lie within this perturbative regime. Using the Taylor expansion for the exponent function, we can write $\hat{S}=\sum_{\phi ,\phi^{\prime }}S_{\phi ,\phi^{\prime }}\left|\phi \right\rangle 〈\left.\phi^{\prime }\right|$ , with

(13) $$S_{\phi ,\phi^{\prime }}=\delta_{\phi \phi^{\prime }}-ih_{I,\phi ,\phi^{\prime }}-\frac{1}{2}\underset{\phi^{\prime \prime }}{\sum }h_{I,\phi ,\phi^{\prime \prime }}h_{I,\phi^{\prime \prime },\phi^{\prime }}+\ldots$$

which shows explicitly the mixing of the states of ${\hat{H}}_{0}$ by the passing electrons to all orders in the coupling strengths given by Equations (4)–(6).

The quasi-analytical character of the approach introduced above provides us with deep insights into the phenomenology of target-probe interactions. In the following sections, we will use it to unveil how the electron-induced state mixing described by Equation (13) can be exploited for the probing of the polaritonic states in our model cavity-QE system. We will focus first on incoming electrons with a well-defined momentum, and then proceed to explore how modulated electron beams can be used to further characterize light–matter SC phenomena through the engineering of the electron wavefunction.

## 4 CL, EELS and PINEM in polaritonic targets

There are two strategies that allow extracting information from the target through the electron probing: through the radiation spectrum of the cavity (we neglect the emission from the QE) into the far-field, as it is done in CL setups, and through the energy lost/gained by the electron beam itself, like in EELS or PINEM experiments. We consider the former first, whose characterization is given by its power spectrum [49]

(14) $$I\left(\omega \right)=\underset{T\to \infty }{\lim}\frac{1}{2\pi T}\overset{\frac{T}{2}}{\underset{-\frac{T}{2}}{\int }}dt\overset{\infty }{\underset{-\infty }{\int }}d\tau \left\langle {\hat{\xi }}^{†}\left(t+\tau \right)\hat{\xi }\left(t\right)\right\rangle {\text{e}}^{-\text{i}\omega \tau },$$

where $\hat{\xi }=\mu_{c_{x,y}}\left({\hat{a}}_{x}+{\hat{a}}_{z}\right)$ is the dipole moment operator of the cavity (describing the coherent light emission [10] by its two degenerate modes) and $\hat{\xi }\left(t\right)={\text{e}}^{\text{i}{\hat{H}}_{0}t/\hbar }\hat{\xi }{\text{e}}^{-\text{i}{\hat{H}}_{0}t/\hbar }$ describes its evolution in time under the bare Hamiltonian in Equation (1). The expectation value in Equation (14) is firstly taken over the state $\left|\phi_{f}\right\rangle =\hat{S}\left|G\right\rangle$ , which results from the fast target-probe interaction when the former is initially in its ground state. We have briefly discussed the lifetime of the target states to justify the approximations inherent to Equation (8). However, our model is based on a purely Hamiltonian description of the target, given by Equation (7). Therefore, the spectrum obtained from Equation (14) will consist of a weighted sum of delta Dirac functions. In the following, we will introduce a phenomenological broadening, *σ*, for the spectral features, to account for the finite lifetime of the target states, by making the replacement $\delta \left(\omega \right)\to \frac{\sigma }{2\pi }\frac{1}{\omega^{2}+\sigma^{2}/4}$ . This Lorentzian lineshape is obtained in the Lindbladian description of open quantum systems [50], [51].

**Figure 3:**
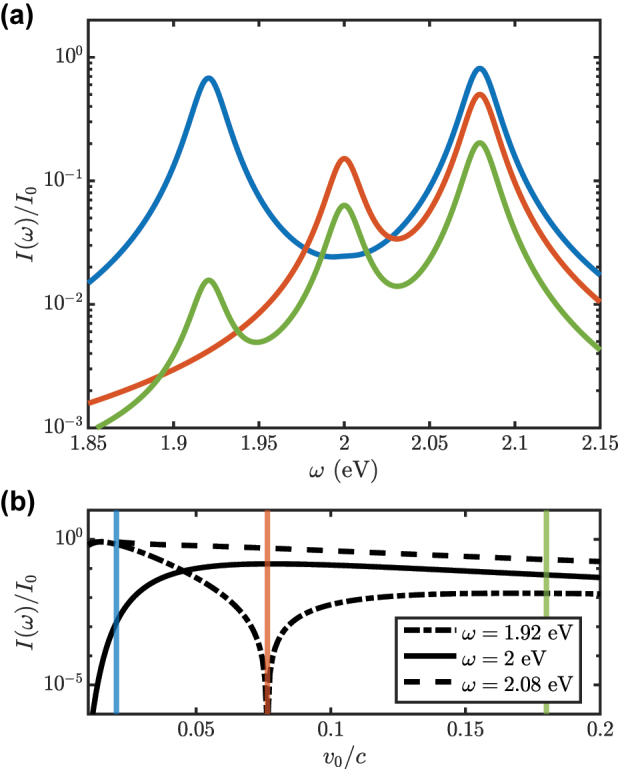
Far-field light intensity versus photon frequency for passing electrons with *b*_*e*−QE_ = 1 nm and *b*_*e*−*c*_ = 11 nm. (a) Power spectra for three different electron velocities, indicated by the vertical arrows in Figure 2: 0.02*c* (blue), 0.08*c* (orange) and 0.18*c* (green). (b) Height of the three maxima in *I*(*ω*) as a function of *v*_0_/*c*. Vertical lines indicate the configurations considered in (a).

Figure 3(a) shows CL-like spectra obtained for aloof electrons with impact parameters *b*_*e*−QE_ = 1 nm, *b*_*e*−*c*_ = 11 nm, and different velocities, indicated by the vertical color arrows in Figure 2. The far-field intensity spectra are broadened by *σ*, set to 0.02 eV, an optimistic estimation for plasmonic lifetimes [48] (1/*σ* = 30 fs). They are normalized to *I*_0_, the intensity at the polariton maxima in the limit *v*_0_ → 0 (see below). Three spectral maxima are apparent, which originate from the upper and lower polaritons, at 2.08 and 1.92 eV, respectively, and the uncoupled *z* − dipole cavity mode at 2 eV. For slow electrons (blue, *v*_0_ = 0.02*c*), the spectrum is dominated by the polariton peaks, which have similar weights. This indicates that the electron-target interaction is mainly taking place through one of the polariton constituents. Indeed, $\left|g_{x}^{e-\text{QE}}|\gg |g_{\omega_{1\pm }/v_{0},x}^{e-c}\right|$ in this case, due to the small value of the QE impact parameter. As expected from Figure 2(c), there is not an intermediate peak in this spectrum, as $g_{\omega_{c}/v_{0},z}^{e-c}$ is negligible in this configuration.

The spectrum for higher electron velocities, *v*_0_ = 0.08*c* (orange), does not present the peak at 1.92 eV, which indicates that the lower polariton has become dark to the incoming electron beam. Note that $g_{x}^{e-\text{QE}}≃g_{\omega_{1\pm }/v_{0},x}^{e-c}$ and *h*_*I*,*G*,−_ vanishes in this case. At even larger velocities, *v*_0_ = 0.18*c* (green), the spectral peaks are, in general, lower, but the three of them are clearly visible. In this configuration, all the matrix elements acquire comparable values. Our results reveal the complex dependence of *I*(*ω*) on *v*_0_/*c*, far from any monotonic trend. In Figure 3(b) we analyze it in more detail, by displaying the height of the CL peaks as a function of the electron velocity. We find that the upper polariton peak is always the largest, while the lower polariton (*z* − dipole mode) is the second largest for low (large) *v*_0_. In the limit *v*_0_ → 0, the upper and lower polariton maxima acquire the same value, *I*_0_, employed for normalization. We can also observe that three far-field intensity maxima approach in the limit of large electron velocity in Figure 3(b). In SM, Section S4, a detailed discussion on the relationship between the peaks of *I*(*ω*) and the electron-target couplings in Equations (4)–(6) is provided.

**Figure 4:**
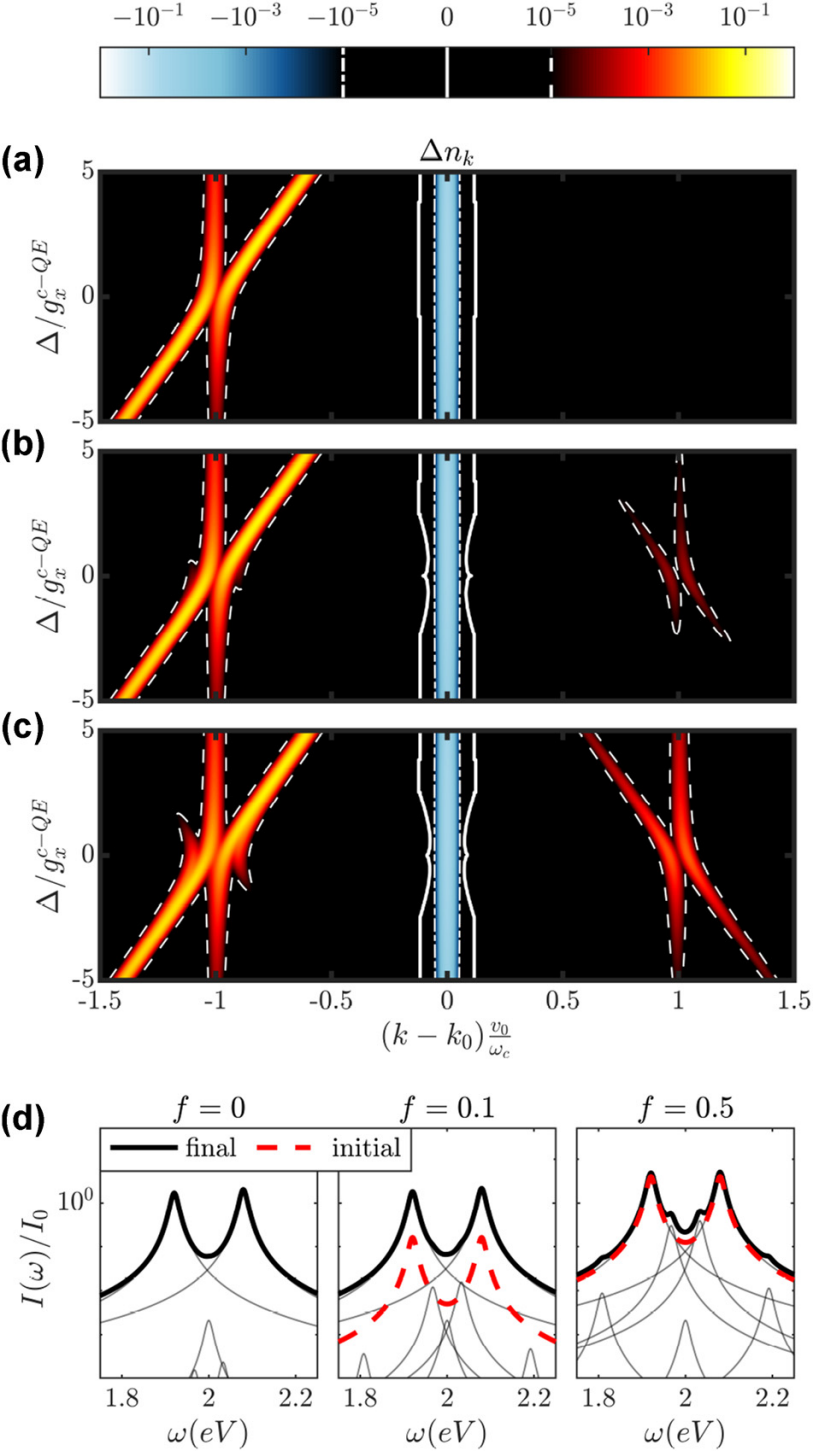
Momentum reshaping experienced by an incident monochromatic electron beam (*k* = *k*_0_, *v*_0_ = 0.02*c*) in its interaction with a polaritonic target as a function of the half cavity-QE detuning Δ = (*ω*_*c*_ − *ω*_QE_)/2. In panel (a), the cavity is initially in its ground state in an EELS-like configuration. In panels (b) and (c), the initial state of the cavity is given by Equation (16) with *f* = 0.1 and 0.5, respectively, mimicking a PINEM setup. Panel (d) shows far-field emission spectra for the targets in the three panels above and Δ = 0. Black solid (red dashed) lines plot the intensity after (before) the interaction with the incoming electrons. Thin grey lines render the different contributions to *I*(*ω*). They correspond to the transitions between the ground state and the $\left|1,\pm \right\rangle$ polaritons and *z*-dipolar optical mode, and between polaritonic states $\left|2,\pm \right\rangle$ and $\left|1,\pm^{\prime }\right\rangle$ .

We investigate next the fingerprint of the target-probe interaction in the wavefunction of the passing electron beam. For this purpose, we focus on the reshaping of the momentum distribution of the electron wavepacket, measured by the difference in the population of the states $\left|k\right\rangle$ before and after the coupling with the QE-cavity system. Expressed in terms of the number operator ${\hat{n}}_{k}={\hat{c}}_{k}^{†}{\hat{c}}_{k}$ , this difference is given by

(15) $$\Delta n_{k}=\left\langle {\hat{n}}_{k}\right\rangle -{\left\langle {\hat{n}}_{k}\right\rangle }^{0}$$

where the superscript 0 indicates that the expectation value is evaluated for the electron wavefunction prior to the interaction. Figure 4(a)–(c) plots this population difference versus $\left(k-k_{0}\right)\frac{v_{0}}{\omega_{c}}$ (where *k*_0_ is the central electron wavevector) and half the detuning between the cavity mode and the QE, Δ = (*ω*_*c*_ − *ω*_QE_)/2 (no longer at resonance in our analysis). The panels correspond to different initial states of the cavity-QE target, parameterized through the variable *f*, the amplitude of the first excited state of the *x*-dipole cavity mode,

(16) $$\left|\phi_{0}\right\rangle ={\left|0\right\rangle }_{z}⊗\left[\sqrt{1-f^{2}}{\left|0\right\rangle }_{x}+f{\left|1\right\rangle }_{x}\right]⊗\left|g\right\rangle ⊗\left|k_{0}\right\rangle .$$

This eigenstate of ${\hat{H}}_{0}$ for vanishing $g_{x}^{c-\text{QE}}$ mimics the weak, coherent driving of the cavity by a laser field polarized along *x*-direction. Note that, we have used the bare basis above, instead of the polaritonic basis employed in the previous section.

In Figure 4(a), we consider an EELS-like configuration, with the target initially in its ground state, $\left|\phi_{0}\right\rangle =\left|G\right\rangle$ (*f* = 0). This setup has been previously investigated in the context of polariton formation in nanophotonic systems [19], [20], [41]. The incoming electron beam is monochromatic, presenting a single wave-vector component, *k* = *k*_0_ and *v*_0_ = 0.02*c* (blue arrow in Figure 2). We can observe that the electron population is transferred to *k* < *k*_0_, the region of energy loss, while, as expected, the energy gain region (*k* > *k*_0_) remains null. At zero detuning, Δ = 0, two maxima in Δ*n*_*k*_ > 0 (yellow color) are apparent, corresponding to the polaritonic states in the first excitation manifold, $\left|1,\pm \right\rangle$ . These emerge in the region $k-k_{0}≃-\frac{\omega_{c}}{v_{0}}$ . The momentum transfer maxima for non-zero detuning disperse, giving rise to the imprint of the anticrossing profile characteristic of light–matter SC [4], [8], [9], [21], [41] into the electron wavepacket. At $\left|\Delta |>|g_{x}^{c-\text{QE}}\right|$ , two asymptotic branches are apparent, one vertical, corresponding to the *x*-dipole cavity mode (fixed *ω*_*c*_), and one diagonal, given by the QE exciton (variying *ω*_QE_). Like in Figure 3, a phenomenological wave-vector broadening *σ*/*v*_0_ has been introduced in the map. The resulting lineshapes are indicated by the solid and dashed lines, which correspond to the isocurves Δ*n*_*k*_ = 0 and |Δ*n*_*k*_| = 10^−5^, respectively.

As shown in Figure 4(b), by pumping weakly the cavity mode (*f* = 0.1), and under a monochromatic electron beam with *k* = *k*_0_ and *v*_0_ = 0.02*c*, a region of Δ*n*_*k*_ > 0 emerges in the energy-gain side of the momentum transfer map. This indicates that, as a result of the interaction with the target, the electron wavepacket can acquire momentum components larger than *k*_0_ thanks to the population in the first excitation manifold of the cavity. This setup mimics a PINEM experiment, in which the passing electrons exchanges energy with an optically-driven resonator. We can observe that the anticrossing profile in the energy-gain region is the fainted mirror image of the energy-loss one, with asymptotic branches given by fixed *ω*_*c*_ and −Δ. At higher driving, *f* = 0.5 in Figure 4(c), the magnitude of the energy gain anti-crossing becomes comparable to its energy loss counterpart, as the amplitude of ${\left|0\right\rangle }_{x}$ and ${\left|1\right\rangle }_{x}$ in Equation (16) are the same. We can also observe extra branches in the energy loss region, that follow −Δ instead of Δ. These Δ*n*_*k*_ maxima originate from the promotion of polaritonic population from the first to the second excitation manifold in the interaction with the passing electrons (discussed in more detail below), and illustrates that the power of PINEM in polaritonic systems for electron wavepacket shaping is well beyond that of EELS.

To complement our study, we plot in Figure 4(d) the emission spectrum calculated from Equation (14) under the driving conditions in panels (a)–(c) and for zero cavity-QE detuning (Δ = 0). Red dashed and black solid lines render *I*(*ω*) (in log scale) before and after the interaction with the electron beam. At *f* = 0 (EELS-CL configuration), *I*(*ω*) = 0 prior to the electron arrival, and the final spectrum is dominated by two maxima originated from the radiative decay of the $\left|1,\pm \right\rangle$ polaritons to the ground state. The lineshapes for these two contributions are rendered in thin grey lines. Due to their lower weight, other spectral contributions also plotted in grey thin lines, are not apparent in *I*(*ω*). The central one corresponds to the *z* − dipole cavity mode (weakly excited by the passing electrons), and the small ones next to it result from the $\left|2,\pm \right\rangle$ to $\left|1,\pm \right\rangle$ transitions, with frequencies $\omega_{2,\pm }-\omega_{1,\pm }=\omega_{c,QE}\pm \left(\sqrt{2}-1\right)g_{x}^{c-\text{QE}}$ .

Figure 4(d) also presents intensity spectra for the two optically-driven cavities in panels (b) and (c), evaluated at *f* = 0.1 and 0.5, respectively. In both cases, the initial spectra present the two main polaritonic peaks only, whose height increases with *f*. In the final *I*(*ω*), multiple contributions can be identified. Apart from the two main ones, whose amplitude barely varies with respect to *f* = 0, and the central *z* − dipole feature which is independent of *f*, we can observe that the weight of the $\left|2,\pm \right\rangle$ to $\left|1,\pm \right\rangle$ transitions grow considerably with increasing optical driving. Moreover, two additional side peaks are apparent, due to another set of second-to-first manifold transitions, $\left|2,\pm \right\rangle$ to $\left|1,\mp \right\rangle$ , with frequencies $\omega_{2,\pm }-\omega_{1,\mp }=\omega_{c,\text{QE}}\pm \left(\sqrt{2}+1\right)g_{x}^{c-\text{QE}}$ . These transitions are also behind the extra branches in the energy-loss side branches of Figure 4(c) at small detuning. Our results evidence that the alteration of *I*(*ω*) due to the passing electron is negligible for cavities under significant optical pumping, a direct consequence of the weak character of the target-probe interaction [38], [39]. Thus, to fully exploit the probing abilities of electron wavepackets, the strength of their coupling to the polaritonic target must be enhanced. In the next section we explore the use of different degrees of freedom of the incoming electron wavefunction for this purpose.

## 5 Modulated electron beams

In the previous section, we have shown that, in a PINEM setup, electron wavefunctions with a rich momentum distribution can be generated from monochromatic electron beams through their interaction with a pumped cavity-QE target. By letting the electrons drift after the interaction, the various momentum components separate in space, giving rise to a series of peaked electron wavepackets. These are usually termed as modulated electron beams. Indeed, in recent years, much research attention have focused on different approaches to generate modulated electron wavefunctions through then interaction with optical systems [52], [53], [54]. Here, we explore the probing capabilities that these modulated electrons bring when interacting with a cavity-QE system, and show that quantum degrees of freedom associated to the electron wavefunction become particularly relevant in the exploration of polaritonic states.

In this section, we will use the same formalism as in the previous one, but for convenience, we will explicitly deal with target and probe degrees of freedom separately. As a starting point, the bare Hamiltonian eigenstates in Equation (7) as $\left|\phi \right\rangle =\left|\varphi_{e}\right\rangle ⊗\left|\psi_{t}\right\rangle$ , where the first (second) wavefunction characterizes the electron beam (target) state. Similarly, the interaction Hamiltonian in Equation (2) can be written as ${\hat{H}}_{I}=\sum_{q}{\hat{H}}_{I,q}\;{\hat{b}}_{q}$ , separating target and electron operators, and the scattering matrix in Equation (8) as

(17) $$\begin{array}{ll} \hat{S} & =exp\left(-i\underset{\psi_{i},\psi_{j}}{\sum }h_{I,\psi_{i},\psi_{j}}|\psi_{i}〉\;〈\psi_{j}|\;{\hat{b}}_{q_{\psi_{i},\psi_{j}}}\right)\\ & =\underset{\psi_{i},\psi_{j}}{\sum }S_{\psi_{i},\psi_{j}}|\psi_{i}〉\;〈\psi_{j}|\;{\hat{b}}_{q_{\psi_{i},\psi_{j}}}, \end{array}$$

with $h_{I,\psi_{i},\psi_{j}}=\frac{L}{\hbar v_{0}}\left\langle \psi_{i}\right|{\hat{H}}_{I,q_{\psi_{i},\psi_{j}}}\left|\psi_{j}\right\rangle$ , and where $q_{\psi_{i},\psi_{j}}=\left(E_{\psi_{i}}-E_{\psi_{j}}\right)/\hbar v_{0}$ is the electron-target momentum exchange (set by energy conservation and the non-recoil approximation). The scattering matrix amplitudes $S_{\psi_{i},\psi_{j}}$ have the same form as Equation (13), but replacing the states $\left|\phi \right\rangle$ by $\left|\psi \right\rangle$ , and the matrix elements *h*_*I*_ by the $h_{I}$ ones above. The structure of the scattering matrix in Equation (17) allows its analytical implementation through the exploitation of the simple algebra of the ${\hat{b}}_{q}$ operators, as detailed in Section S6 of the SM. This is where the power of our approach resides, as it makes it possible to obtain analytical expressions for the observables of interest.

We consider now a modulated electron beam, initially prepared in a superposition of momenta of the form $\left|\varphi_{e}\right\rangle =\sum_{k}B\left(k\right)\left|k\right\rangle$ (with ∑_*k*_|*B*(*k*)|^2^ = 1). This wavefunction can describe, for instance, a comb with a set of amplitude peaks equally spaced in momentum space. We assume that the target is prepared initially in one of its polaritonic eigenstates, $\left|\psi_{m}\right\rangle ={\left|n\right\rangle }_{z}⊗\left|N,\pm \right\rangle$ . If, for convenience, we employ its density matrix description of the target, we have $\rho_{t}^{0}=\left|\psi_{m}\right\rangle \left\langle \psi_{m}\right|$ . After the interaction with the modulated electrons, it reads

(18) $$\begin{array}{ll} \rho_{t} & =\underset{k}{\sum }\left\langle k\right|\hat{S}\left|\varphi_{e}\right\rangle \rho_{t}^{0}\left\langle \varphi_{e}\right|{\hat{S}}^{†}\left|k\right\rangle \\ & =\underset{\psi_{i},\psi_{j}}{\sum }S_{\psi_{i},\psi_{m}}S_{\psi_{j},\psi_{m}}^{*}|\psi_{i}〉\;〈\psi_{j}|\\ & \;\times \left[\underset{k}{\sum }B\left(k\right)B^{*}\left(k-q_{\psi_{i},\psi_{j}}\right)\right]. \end{array}$$

The expression above shows that the population of the target states are completely independent from the electron momentum distribution [37], as $\left\langle \psi_{s}\right|\rho_{t}\left|\psi_{s}\right\rangle =|S_{\psi_{s},\psi_{m}}{|}^{2}$ . Furthermore, $\left\langle \psi_{s}\right|\rho_{t}\left|\psi_{s}\right\rangle ≃\left\langle \psi_{s}\right|\rho_{t}^{0}\left|\psi_{s}\right\rangle =\delta_{s,m}$ ( $S_{\psi ,\psi }=1$ to first order in the electron-target interaction), which shows that initial populations remain largely unchanged after the interaction with the electron. On the contrary, the coherences can be manipulated by appropriately designing the electron wavefunction [37]. Thus, by shaping *B*(*k*) as a finite momentum comb with spacing $q_{\psi_{m_{1}},\psi_{m_{2}}}$ , the coherence $\left\langle \psi_{m_{1}}\right|\rho_{t}\left|\psi_{m_{2}}\right\rangle$ will be modified, while leaving the rest of the target density matrix unaltered.

Next, we focus our attention on targets prepared in a superposition of polaritonic states of the form $\left|\psi_{t}^{0}\right\rangle =\cos\phi \left|\psi_{m_{1}}\right\rangle +{\text{e}}^{\text{i}\theta }\sin\phi \left|\psi_{m_{2}}\right\rangle$ . Then, the population of a given polaritonic state $\left|\psi_{s}\right\rangle$ after interaction with the modulated electron beam has the form

(19) $$\begin{array}{ll} \left\langle \psi_{s}\right|\rho_{t}\left|\psi_{s}\right\rangle & =\cos^{2}\phi {\left|S_{\psi_{s},\psi_{m_{1}}}\right|}^{2}+\sin^{2}\phi {\left|S_{\psi_{s},\psi_{m_{2}}}\right|}^{2}\\ & \;+\text{Re}\left\{{\text{e}}^{-\text{i}\theta }\sin2\phi \;S_{\psi_{s},\psi_{m_{1}}}S_{\psi_{s},\psi_{m_{2}}}^{*}\right.\\ & \left.\;\times \underset{k}{\sum }B\left(k\right)B^{*}\left(k-q_{\psi_{m_{2}},\psi_{m_{1}}}\right)\right\}, \end{array}$$

which explicitly shows that for arbitrary initial target state, the final polaritonic populations can vary thanks to the coherences in $\rho_{t}^{0}$ (before the target-probe interaction) and the modulation of the electron beam encoded in *B*(*k*). See more details in Section S6 of the SM. The last term indicates that by targeting the transition between the polaritonic states involved in $\left|\psi_{t}^{0}\right\rangle$ , the impact of the modulation on the populations can be maximized. This ability (showcased here for a general target) of modulated electrons to transform coherences into populations is what enables them to induce Rabi dynamics in QEs [38] and makes it possible using them to implement quantum state tomography protocols [39].

To illustrate the implications of Equation (19), we consider a particular target-probe configuration. The initial electron wavefunction is set to a comb of the form $\left|\psi_{e}\right\rangle =\sum_{n=-N/2}^{N/2}\frac{\left|k_{0}+nq_{mod}\right\rangle }{\sqrt{N+1}}$ with *N* = 100. Note that it implies the exchange of up to 50 photons in its preparation (well within reach of recent PINEM experiments [55]). The polaritonic target is initially in the state

(20) $$\left|\psi_{t}^{0}\right\rangle =\frac{1}{2}{\left|0\right\rangle }_{z}⊗\left[\sqrt{3}{\left|0\right\rangle }_{x}+{\text{e}}^{\text{i}\theta }{\left|1\right\rangle }_{x}\right]⊗\left|g\right\rangle ,$$

where *θ* is a real number. Note that this wavefunction can be expressed as a linear combination of the target ground state and the ${\left|0\right\rangle }_{z}⊗\left|1,\pm \right\rangle$ polaritonic states.

**Figure 5:**
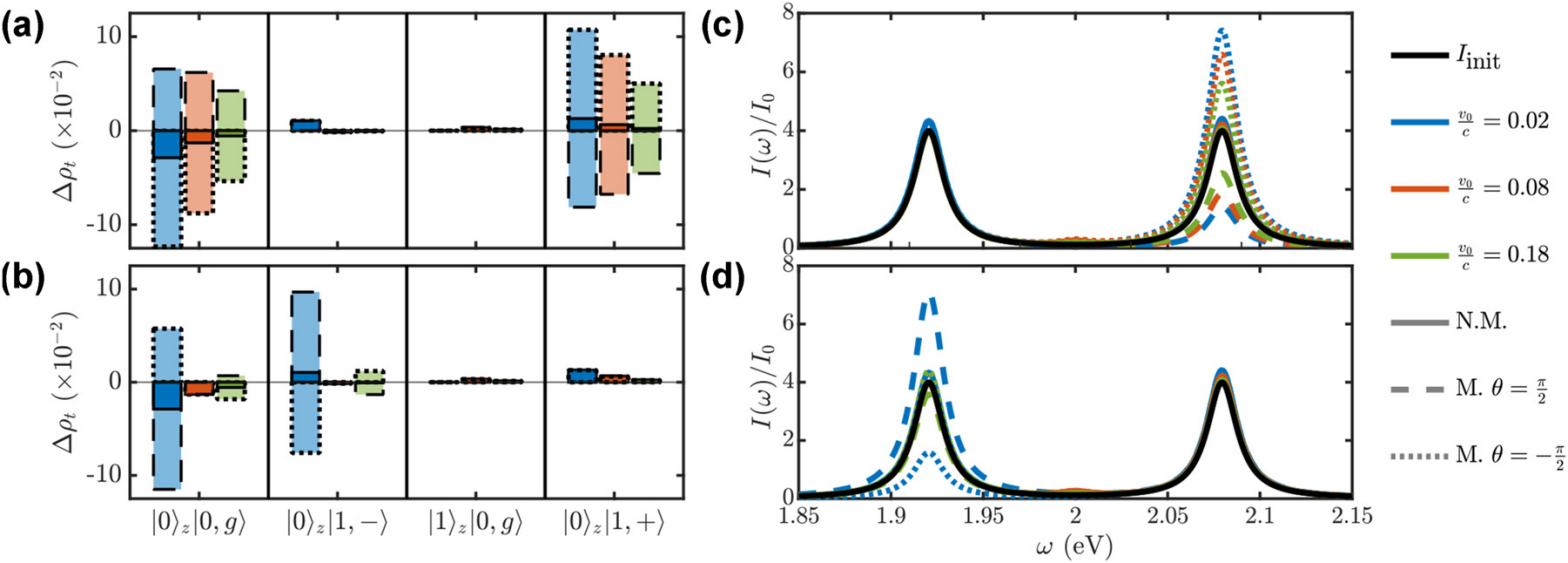
Impact of the electron modulation on the target population transfer (a)–(b) and cavity power spectrum (c)–(d). In the top (bottom) panels, the momentum modulation is at resonance with the transition between the ground state and upper (lower) polariton state, *q*_mod_ = *ω*_1,+_/*v*_0_ (*q*_mod_ = *ω*_1,−_/*v*_0_). Three different electron central velocities are considered: 0.02*c* (blue), 0.08*c* (orange) and 0.2*c* (green), and the impact parameter *b*_*e*−*c*_ is set to 11 nm. In all panels, two different initial state phases, *θ* are considered: *π*/2 (dashed lines) and −*π*/2 (dotted lines). The solid lines correspond to a non-modulated (N.M.) electron beam, and the solid black lines in (c) and (d) plot the cavity spectrum before the interaction with the passing electrons.

In Figure 5(a) and (b), we analyze the population differences (given by the diagonal terms of $\Delta \rho_{t}=\rho_{t}-\rho_{t}^{0}$ ) induced by the passing electrons on the ground and the three first-excitation target states. We consider the three central electron velocities indicated in Figure 2 (blue, orange and green in increasing *v*_0_/*c*), and two different initial state configurations, given different values of *θ* in Equation (20) (dashed and dotted lines). In all cases, *b*_*e*−*c*_ = 11 nm. For reference, the population differences for a non-modulated (N.M.) electron beam are plotted in solid lines (note that these are independent of *θ*). In panel (a), the modulation spacing is at resonance with the upper polariton, *q*_mod_ = *ω*_1,+_/*v*_0_, in panel (b), with the lower one, *q*_mod_ = *ω*_1,−_/*v*_0_.

As expected, Figure 5(a) displays a significant population transfer only between the ground state (left) and the upper polariton (right), which is larger for lower electron velocity, following the monotonic dependence in the emitter-target coupling in Figure 2(a). Moreover, we can observe that for *θ* = −*π*/2 the upper polariton gains population (as in the non-modulated case), while it gets depopulated for *θ* = *π*/2. Note that this parameter sets the phase, and therefore the sign, of the contribution of the initial coherences to the final populations given by the last term in Equation (19). We can see how this can be leveraged to control the flow of population among polaritonic states. The momentum spacing in *B*(*k*) is set to yield the most efficient energy transfer between the ground and lower polariton states in Figure 5(b). The non-monotonic dependence of the populations on the electron velocity in this case is inherited from Figure 2(b). Again, varying *θ* inverts the direction of the population transfer.

Apart from analyzing the effect of electron modulation on the target populations, we also investigate its impact on the cavity power spectrum given by Equation (14), now evaluated for the state that results from applying the scattering matrix on Equation (20). Importantly, this is a far-field magnitude that can be easily accessed experimentally. Figure 5(c) and (d) plot *I*(*ω*) for *q*_mod_ = *ω*_1,+_/*v*_0_, and *q*_mod_ = *ω*_1,−_/*v*_0_, respectively. The black solid line renders the cavity spectrum before the interaction with the electron beam, *I*_init_. We can observe that only the upper polariton peak is shaped by the passing electrons in (c), and the lower polariton one in (d). This illustrates the far-field fingerprint of the population manipulation in panels (a) and (b). In both cases, only the emission from the targeted transition through *q*_mod_ is modified, keeping the spectrum around the other features unaltered. Importantly, as we observed in the polariton populations, the initial coherences, whose contribution to the spectrum depends on *θ*, set whether the altered emission peak increases or decreases with respect to *I*_init_.

Figure 5 indicates that the coherences, rather than the populations, in $\rho_{t}^{0}$ dictate the manner in which the population transfer and the spectrum reshaping take place through the interaction with the modulated electron beam. To gain insight into this result, we simply evaluate Equation (19) for polaritonic states that are initially populated, i.e., by making *s* = *m*_1_, for example. It is then straightforward to see then that, in the first two terms, $|S_{\psi_{m1},\psi_{m1}}{|}^{2}=1$ and $|S_{\psi_{m1},\psi_{m2}}{|}^{2}={|h}_{I,\psi_{m1},\psi_{m2}}{|}^{2}$ to first order in the electron-target interaction. On the contrary, we have $S_{\psi_{m1},\psi_{m1}}S_{\psi_{m1},\psi_{m2}}^{*}{=h}_{I,\psi_{m1},\psi_{m2}}$ to first order in the last one. Thus, we find that, while the first terms are independent or quadratic on the electron-target interaction strength, the last is linear, which makes it the leading one. Moreover, it is easy to show that ∑_*k*_ *B*(*k*)B*(*k* − *q*_mod_) = *N*/(*N* + 1) ≃ 1 for a finite, but long, electron comb, also contributing to make the initial coherences crucial in establishing the effect of the passing electrons on the target. Note that adding a running phase difference between the different momentum peaks of the initial electronic wavefunction as $\left|\psi_{e}\right\rangle =\sum_{n=-N/2}^{N/2}{\text{e}}^{\text{i}n\xi }\frac{\left|k_{0}+nq_{mod}\right\rangle }{\sqrt{N+1}}$ yields an extra phase factor in the modulated contributions of Equation (19), which allows to externally control the internal dynamics of the target.

**Figure 6:**
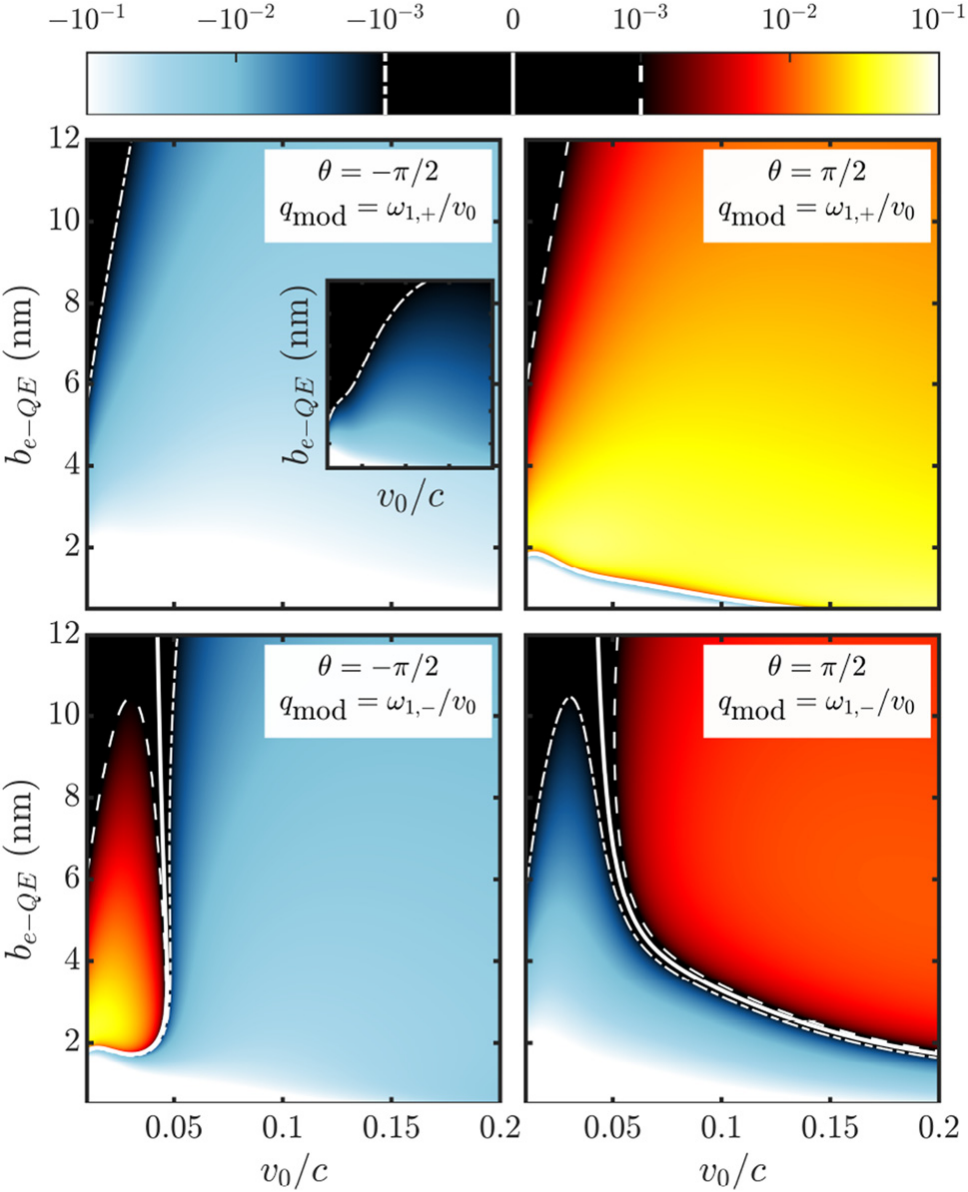
Expectation value of the energy change of the electron in units *ℏω*_*c*_. Main panels show the case of modulated electrons, showcasing that net energy gain and loss is achievable by modulating the electron. On each panel we show the modulation spacing and also the phase factor of the initial target state. The inset corresponds to the case of a non-modulated electron, where there always is net energy loss. The result in this case is independent of the phase factor.

Finally, we pay attention to the effect that the target-probe interaction has on the modulated electron beam. The fact that the population transferences induced by modulated beams are larger than the non-modulated ones means that the energy balance of the interaction can be altered through the modulation itself. Thus, it is possible, in principle, to pump or deplete the target. In Figure 6, we explore the net energy change experienced by the passing electrons

(21) $$\Delta E=\underset{k}{\sum }E_{k}\Delta n_{k},$$

where Δ*n*_*k*_ is defined in Equation (15). As the initial electron wavefunction, we take the finite comb in Figure 5 and the target is prepared in the state given by Equation (20).

The four panels in Figure 6 display Δ*E* in units of *ℏω*_*c*_ as a function of the central electron velocity and impact parameter, *b*_*e*−QE_. The results for the initial target state with *θ* = −*π*/2 (*θ* = *π*/2) are shown in the left (right) maps, and the modulation is set at resonance with the ground transition to the upper (top) and lower (bottom) polariton. For reference, the map for non-modulated electrons is shown as an inset with the same parameter range, illustrating that the passing electrons can only lose energy in the non-modulated setup, and Δ*E* is larger for smaller impact parameter and electron velocity. The situation is rather similar for *θ* = −*π*/2. For this state phase, there emerges only a narrow region of small *v*_0_/*c* where the electron beam gains energy for *q*_mod_ = *ω*_1,−_/*v*_0_. Apart from it, the maps resemble the EELS one, and the target populations always increases by the effect of the passing electrons. For *θ* = *π*/2 (right), the net energy change maps are very different. Fast electron beams gain energy for both *q*_mod_ (although Δ*E* is larger for the transition between the ground and upper polariton), and lose it at low velocities and impact parameters. Here, the target is populated/depopulated depending on *v*_0_ and *b*_*e*−QE_. The richness of the net energy loss/gain landscape in Figure 6 follows from the coupling strengths in Figure 2, as the leading order in the electron-target interaction is linear in *h*_*I*_. Thus, we can link the gain-loss transitions in the lower maps with the change in sign in *h*_*I*,*G*,1−_ in Figure 2(b). All maps are equivalent in the limit of small *v*_0_ and *b*_*e*−QE_, as *h*_*I*_ → 1 in this limit and the electron modulation becomes irrelevant. Our results also showcase the power of polaritonic systems to re-shape and alter modulated electron beams through the energy of its natural transitions and the phase involved in its initial state preparation.

## 6 Conclusions

We have presented a comprehensive study of the probing of polaritonic systems by electron beams. The target is composed by a nanophotonic cavity supporting two dipolar modes, and a quantum emitter strongly coupled to one of them. Using macroscopic QED, we have built a model Hamiltonian describing the interaction between probe and target, fully parameterized in terms of the dyadic Green’s function in the quasi-static approximation. We have analyzed the effect of electron–polariton interactions on different observables, including the electron momentum distribution and net energy change, and the polaritonic state populations and the light emission spectrum by the target. Our investigation has proceeded by increasing the complexity on the electron beam and target preparation, from EELS and CL to PINEM, and finally PINEM with modulated electron beams. All these described using the same, unifying theoretical model. Our results show that free electrons, through the modulation of their wavefunction, are a powerful probe, and also a suitable tool for the manipulation, of quantum targets with a complex energy ladder of (bright and dark) excitations, such as polaritonic systems.

## Supplementary Material

Supplementary Material Details
