# Supplementary material for: Electron-assisted probing of polaritonic light–matter states
Source: Nanophotonics. 2024 Feb 27;13(11):2015–27. doi: 10.1515/nanoph-2023-0907 (PMC11501223; doi:10.1515/nanoph-2023-0907)
Supplement: Supplementary file 1 — Supplementary Material Details [file j_nanoph-2023-0907_suppl_001.pdf]

J. Abad-Arredondo* and A. I. Fernández-Domínguez*

Supplementary Material: Electron-assisted probing of polaritonic light-matter states

Abstract: This Supplementary Material provides details on different aspects of the theoretical approach presented in the main text. Section S1 showcases a general overview of the construction of the Hamiltonian. Sections S2 to S4 describe the individual steps taken in its development. Section S2 extends previous frameworks for the study the field-assisted interaction between generic electronic transitions, starting from the Macroscopic QED formalism. Section S3 applies the ideas of emitter-centered modes to define physically meaningful bosonic operators for these general electronic transitions. In Section S4, we particularize the general couplings obtained in previous sections to the interaction with a metallic nanoparticle, by deriving its Dyadic Green's function in the quasi-static limit. A general calculation of the final populations of any quantum target after the interation with a modulate electron are given in Section S6, and Section S7 provides insight into the symbolic computational implementation of our calculations.

S1 Outline of the hamiltonian derivation

In this section, we give an overview of the way the hamiltonian of the system has been postulated. For a more detailed description of the followed procedure we refer the reader to the corresponding sections of this Supplementary Material document. The starting point is a Macroscopic QED (MQED) hamiltonian describing EM fields in a minimal coupling scheme and a term that describes a collection of arbitrary electronic eigenstates:

$$\hat{H} = \sum_\lambda \int\int dr\, d\omega\, \hbar\omega \hat{f}_\lambda^\dagger(r,\omega)\hat{f}_\lambda(r,\omega) + \sum_i E_i \hat{c}_i^\dagger \hat{c}_i + \frac{e}{m}\hat{p}\cdot\hat{A}, \tag{s1}$$

where $f_\lambda(r,\omega)$ are the usual bosonic operators representing the fields created by an infinitesimal dipole moment, $\hat{c}_i$ is the annihilation operator of an electron in an eigenstate described by the wavefunction $\phi_i(r)$, and $\hat{p}$ and $\hat{A}$ are the usual momentum and vector potential operators. In the above hamiltonian, all the interactions between different electronic transitions are mediated by EM fields, and as such these interactions are a second order process. The first step is to derive an effective hamiltonian that explicitly couples different electronic transitions. Following a procedure similar to [1] (described in Section S2.1) we find that the dynamics of the system can be described by an effective hamiltonian

$$\hat{H}_{\text{eff}} = \sum_\lambda \int\int dr\, d\omega\, \hbar\omega \hat{f}_\lambda^\dagger(r,\omega)\hat{f}_\lambda(r,\omega) + \sum_i (E_i - \hbar\delta_i)\, \hat{c}_i^\dagger \hat{c}_i + \frac{e}{m}\hat{p}\cdot\hat{A}' - \hbar \sum_{\substack{i,j\\k,l}} g_{ij,kl}^{m-m} \sigma_{ij}\sigma_{kl}^\dagger \tag{s2}$$

where $\sigma_{ij} = \hat{c}_i^\dagger \hat{c}_j$ is an operator describing the electronic transition $\phi_j \to \phi_i$, and the expressions for the Lamb-shift, δ_i and coupling between electronic transition $g_{ij,kl}^{m-m}$ are given by

$$g_{ij,kl}^{m-m} = \tfrac{e^2\hbar\mu_0}{2m^2} \times \iint d^3r\, d^3 \quad r'\, \vec{d}_{ij}(r)\cdot \text{Re}\left\{\overline{\overline{G}}(r,r',\Omega)\right\}\cdot \vec{d}_{kl}^*(r'), \tag{s3}$$

$$\delta_i = \textstyle\sum_j g_{ij,ij}^{m-m}, \tag{s4}$$

***Corresponding author: J. Abad-Arredondo,** Departamento de Física Teórica de la Materia Condensada and Condensed Matter Physics Center (IFIMAC), Universidad Autónoma de Madrid, E- 28049 Madrid, Spain, jaime.abad@uam.es https://orcid.org/0000-0003-3980-966X (JAA)

***Corresponding author: A. I. Fernández-Domínguez,** Departamento de Física Teórica de la Materia Condensada and Condensed Matter Physics Center (IFIMAC), Universidad Autónoma de Madrid, E- 28049 Madrid, Spain, a.fernandez-dominguez@uam.es https://orcid.org/0000-0002-8082-395X (AIFD)

where $\vec{d}_{ij}(r) = \phi_i^*(r)\nabla\phi_j(r)$ is a dipole moment density that comes from expanding the momentum operator in the basis of electronic eigenstates and $\overline{\overline{G}}(r,r',\Omega)$ is the classical dyadic Green's function of the EM problem. One of the assumptions needed for the above expression to hold is that the energy difference associated with transitions $i \to j$ and $k \to l$ are similar so that $\Omega = \omega_{ij} \approx \omega_{kl}$. This effective hamiltonian would also hold in the case that the Greens function varies sufficiently smoothly over the frequency range of interest. Note that since $\vec{d}_{ij}^* = -\vec{d}_{ji}$, and the dyadic is symmetric with respect to index exchange, it can be shown that this coupling satisfies: $g_{ij,kl}^{m-m} = (g_{kl,ij}^{m-m})^*$ and $g_{ij,kl}^{m-m} = g_{lk,ji}^{m-m}$.

Once that we have included a direct field-mediated coupling between electronic transitions, we proceed to introduce new bosonic operators describing the EM field in the spirit of the emitter-centered modes described in [2]. This is done in Section S2.2, and the final hamiltonian reads

$$\begin{aligned}\hat{H}_{\text{eff}} = \sum_{ij}\int d\omega\, \hbar\omega\, \hat{a}_{ij}^\dagger(\omega)\hat{a}_{ij}(\omega) + \sum_i (E_i - \hbar\delta_i)\,\hat{c}_i^\dagger\hat{c}_i \\ +\hbar\sum_{ij}\int d\omega\, g_{ij}^{l-m}(\omega)\left[\hat{a}_{ij}(\omega) + \hat{a}_{ji}(\omega)^\dagger\right]\sigma_{ij} \\ -\hbar\sum_{\substack{i,j\\k,l}} g_{ij,kl}^{m-m}\sigma_{ij}\sigma_{kl}^\dagger,\end{aligned} \tag{s5}$$

where the new set of bosonic operators $\hat{a}_{ij}$ is defined as

$$\hat{a}_{ij}(\omega) = \frac{-e}{m\omega g_{ij}^{l-m}(\omega)}\iint d\mathbf{r}\, d\mathbf{r}'\, \vec{d}_{ij}(\mathbf{r})\cdot\left(\sum_\lambda \overline{\overline{G}}_\lambda(\mathbf{r},\mathbf{r}',\omega)\cdot\hat{f}_\lambda(\mathbf{r}',\omega)\right), \tag{s6}$$

and satisfy the canonical conmutation relation of bosonic operators by construction: $[\hat{a}_{ij}(\omega), \hat{a}_{ij}^\dagger(\omega)] = 1$, which leads to the following expression for the couplings

$$g_{ij}^{l-m}(\omega) = \frac{e}{m}\sqrt{\frac{\hbar\mu_0}{\pi}}\sqrt{\iint dr\, dr'\, \vec{d}_{ij}(r)\cdot \text{Im}\Big\{\overline{\overline{G}}(r,r',\omega)\Big\}\cdot\vec{d}_{ij}^*(r')}. \tag{s7}$$

Since the bosonic operators are constructed to comply with canonical conmutation relations, the couplings that derive from these are always physical, and independent of the particular system under treatment. We remark that until this point, no knowledge about the particular electronic transitions under study have been assumed, and therefore this treatment can be applied to a wide variety of systems beyond the present one. In Section S3, we find that the current densities,$\vec{d}_{ij}(r)$, associated to transitions in dipolar QE and free electrons propagating along the $\hat{z}$ axis are given by:

$$\vec{d}_{ij}^{QE}(\mathbf{r}) = -\frac{m\omega_{ij}}{e\hbar}\vec{\mu}_{ij}\delta^3(\mathbf{r}-\mathbf{r}_0), \tag{s8}$$

$$\vec{d}_{ij}^{e}(\mathbf{r}) = ik_0\frac{\delta^2(\mathbf{r}-\mathbf{r}_\perp)}{L}e^{i(k_j-k_i)z}\hat{z}, \tag{s9}$$

where $\vec{\mu}_{ij} = -e\langle j|\,\hat{r}\,|i\rangle$ is the usual transition dipole moment, $k_0 = mv_0/\hbar$ is the initial momentum of the incoming electron and L is the length of a fictitious box used to quantise the momentum values of the electron. We have also introduced $\mathbf{r}_0$ as the location of the dipolar QE and $\mathbf{r}_{\perp,0}$ for the location of the free electron on the $x-y$ plane. To obtain the light-matter interaction coupling strengths, we derive the Green's function for a metallic sphere in Section S4, and noting that it can be written as $\overline{\overline{G}}(\mathbf{r},\mathbf{r}',\omega) \approx \overline{\overline{\mathcal{G}}}(\mathbf{r},\mathbf{r}',\omega_C)\delta(\omega-\omega_C)$, where ω_C is the resonance frequency of the cavity, the coupling strength and interaction hamiltonian for dipolar QE interacting with a single cavity mode are given by:

$$\hat{H}_I^{c-QE} = \hbar g^{c-QE}\left[\hat{\sigma}\hat{a}^\dagger + \hat{\sigma}^\dagger\hat{a}\right], \tag{s10}$$

$$g^{c-QE} = \frac{|\omega_{QE}|}{\hbar}\sqrt{\frac{\hbar\mu_0}{\pi}\boldsymbol{\mu}\cdot\text{Im}\left\{\overline{\overline{\mathcal{G}}}(\mathbf{r}_0,\mathbf{r}_0,\omega_c)\right\}\cdot\boldsymbol{\mu}^*}. \tag{s11}$$

The spherical nanoparticle supports three degenerate dipolar modes that can be considered to be oriented along the $\hat{x}$, $\hat{y}$ and $\hat{z}$ axis respectively. The coupling between a QE, placed along the $\hat{x}$ axis to each of these degenerate modes is given in Equations s67-s69. In the case we are considering, with the dipole moment of the QE's transition oriented along the $\hat{x}$ axis, then only the coupling to the $\hat{x}$-mode of the cavity is non-zero and is given by:

$$g_x^{c-QE} = \frac{|\omega_{QE}|}{3}\sqrt{\frac{\pi}{2}\left(\frac{R}{b_{c-QE}}\right)^3 \frac{1}{\hbar\omega_c}\frac{\mu_{QE}^2}{\epsilon_0\, b_{c-QE}^3}}.$$

On the other hand, the hamiltonian describing the coupling between free electrons and the cavity is given by

$$\hat{H}_I^{e-c} = \hbar\sum_q g_q^{e-c}\hat{b}_q\left(\hat{a}^\dagger - \hat{a}\right)\mathrm{sign}(q), \tag{s12}$$

$$g_q^{e-c} = \frac{ek_0}{mL}\sqrt{\frac{\hbar\mu_0}{\pi}\iint dz\, dz'\ \mathrm{Im}\left\{\hat{z}\cdot\overline{\overline{\mathcal{G}}}(\mathbf{R},\mathbf{R}',\omega_c)\cdot\hat{z}\right\}e^{iq(z-z')}}, \tag{s13}$$

where for compactess we have defined $\mathbf{R} = [\mathbf{r}_{\perp,0}, z]$ and $\mathbf{R}' = [\mathbf{r}_{\perp,0}, z']$. Introducing the GF of the cavity we find that the free-electron-cavity couplings can be written as

$$g_{q,x}^{e-c} = \frac{e\hbar k_0}{3mL}|q|^2 K_1(|qb_{e-c}|)\sqrt{\frac{1}{\epsilon_0\hbar\omega_c}\frac{\pi R^3}{2}}, \tag{s14}$$

$$g_{q,y}^{e-c} = 0, \tag{s15}$$

$$g_{q,z}^{e-c} = \frac{e\hbar k_0}{3mL}|q|^2 K_0(|qb_{e-c}|)\sqrt{\frac{1}{\epsilon_0\hbar\omega_c}\frac{\pi R^3}{2}}, \tag{s16}$$

which shows that the $\hat{y}$ dipolar mode of the cavity is decoupled from the electron and QE and therefore remains outside of the dynamics, while the $\hat{x}$ mode couples to free-electron and QE and the $\hat{z}$ mode couples only to the free electron.

Finally, the interaction between the free electron and dipolar transition inside the QE can be described through the matter-matter interaction term in the MQED hamiltonian. Upon introducing the particular definition of the current densities associated to both transitions, the interaction hamiltonian and coupling strength are given by:

$$\hat{H}_I^{e-QE} = -\hbar\sum_q g_q^{e-QE}\left[\hat{\sigma} - \hat{\sigma}^\dagger\right]\hat{b}_q, \tag{s17}$$

$$g_q^{e-QE} = ik_0\frac{e\mu_0\omega_{QE}}{mL}\int dz'\,\boldsymbol{\mu}\cdot\mathrm{Re}\left\{\overline{\overline{G}}\left(\mathbf{r}_0,\mathbf{R}',|\omega_{QE}|\right)\right\}\cdot\hat{z}e^{iqz'}. \tag{s18}$$

Upon realizing that this coupling strength depends on the real part of the GF, and that in resonance the cavity's GF is purely imaginary, then the QE-free electron interaction will be mediated by the vacuum's GF. Upon integration the coupling for an arbitrary dipole orientation is given by Equation s63. Particularizing for a QE oriented along the $\hat{x}$ direction yields a coupling strength:

$$g_q^{e-QE} = -\frac{e\,k_0\,|q|^2}{2\pi mL\epsilon_0\omega_{QE}}\mu_{QE}\mathrm{sign}(qb_{e-QE})K_1(|qb_{e-QE}|) \tag{s19}$$

Putting all these interaction terms together, and introducing the energies for the bare optical modes $\hbar\omega_c\hat{a}_x^\dagger\hat{a}_x$, $\hbar\omega_c\hat{a}_z^\dagger\hat{a}_z$, electronic eigenstates $\sum_i E_i\hat{c}_i^\dagger\hat{c}_i$ and bare QE energies $\hbar\omega_{QE}\hat{\sigma}^\dagger\hat{\sigma}$ we have the complete hamiltonian from the main text.

To end this section, we remark that the interaction between a free electron and optical cavity and QE have been studied before (mostly as separate processes, but recently also in conjunction), and their coupling parametrized. However, in this work we have managed to unify their description in terms of the common starting framework of MQED. This ensures proper normalization of optical modes in arbitrary EM

environments, and therefore, physically meaningful coupling stregths, even in situations in which the EM environment only serves as a non-resonant background. This work paves the way for exploring quantum phenomena in field mediated interactions between arbitrary electronic transitions, and in general light matter interaction phenomena beyond the dipolar approximation of quantum emitters.

S2 General light matter interaction hamiltonian for arbitrary electronic transitions

S2.1 Matter-matter interaction through light

From a minimal coupling scheme within a MQED formalism, one is able to describe the coupling between electronic transitions and field photons quite easily. This in particular means that the field-mediated interactions between electronic transitions become a second order process. In what follows, we would like to deal with an effective hamiltonian, in which the field mediated interaction between different electronic transitions is treated as a first order term. For that, we follow the same stratgy as reference [1], but generalise the formalism for an arbitrarily big set of electronic states. Most of the algebra done here mirrors that of said reference, and we refer the reader there for additional indications on how to perform the derivation. We start from the hamiltonian in a minimal coupling scheme [3], by adapting the Coulomb gauge and neglecting the ponderomotive interaction term of the field, the minimal coupling hamiltonian can be written as:

$$\hat{H} = \boxed{\sum_\lambda \iint d\mathbf{r}\, d\omega\, \hbar\omega \hat{f}^\dagger_\lambda(\mathbf{r},\omega)\hat{f}_\lambda(\mathbf{r},\omega)} \quad + \quad \boxed{\frac{\hat{p}^2}{2m} + V(\mathbf{r})} \quad + \quad \boxed{\frac{e}{m}\hat{p}\cdot\hat{A}} \tag{s20}$$

where we have boxed the terms corresponding respectively to the field hamiltonian ($\hat{H}_F$), a term for the electronic eigenstates ($\hat{H}_e$), and the coupling term ($\hat{H}_{e,F}$). We have introduced the $\hat{f}$ operators, as defined in [3], which act as elemental field excitations, and follow bosonic conmutation relations:

$$[\hat{f}_\lambda(\mathbf{r},\omega), \hat{f}_{\lambda'}(\mathbf{r}',\omega')] = [\hat{f}^\dagger_\lambda(\mathbf{r},\omega), \hat{f}^\dagger_{\lambda'}(\mathbf{r}',\omega')] = \mathbf{0}, \tag{s21}$$

$$[\hat{f}_\lambda(\mathbf{r},\omega), \hat{f}^\dagger_{\lambda'}(\mathbf{r}',\omega')] = \delta_{\lambda,\lambda'}\delta(\mathbf{r}-\mathbf{r}')\delta(\omega-\omega'). \tag{s22}$$

Through the electronic hamiltonian, we obtain the electronic wavefunctions that fulfill $\hat{H}_e\phi_i = E_i\phi_i$, and use these as a basis for the electronic states. As such we can write the electronic term as

$$\hat{H}_e = \sum_i \hat{c}^\dagger_i \hat{c}_i E_i \tag{s23}$$

where $\hat{c}_i$ is the annihilation operator of the state ϕ_i. In the same way, we may introduce these wavefunctions in the interaction term:

$$\begin{aligned}\hat{p}\cdot\hat{A} =& -i\hbar\sum_{i,j}\hat{c}^\dagger_i\hat{c}_j\int d\mathbf{r}\,\phi^*_i(\mathbf{r})\nabla\phi_j(\mathbf{r})\cdot\hat{A}(\mathbf{r}) = \\ =&\frac{-i\hbar}{2}\sum_{i,j}\left[\hat{c}^\dagger_i\hat{c}_j\left(\int d\mathbf{r}\,\phi^*_i(\mathbf{r})\nabla\phi_j(\mathbf{r})\cdot\hat{A}(\mathbf{r})\right) - \left(\int d\mathbf{r}\,\hat{A}(\mathbf{r})\cdot\phi_i(\mathbf{r})\nabla\phi^*_j(\mathbf{r})\right)\hat{c}^\dagger_j\hat{c}_i\right]\end{aligned} \tag{s24}$$

where we have expressed the interaction term in a manifestly hermitian way. We define $\hat{\sigma}_{ij} = \hat{c}^\dagger_i\hat{c}_j$ and $\mathbf{d}_{ij}(\mathbf{r}) = \phi^*_i(\mathbf{r})\nabla\phi_j(\mathbf{r})$. Note that the main difference between this work and reference [1] is that we are allowing for arbitrary electronic transitions mediated by photons to take place, which leads to the natural apparition of these extended transition dipole densities $\mathbf{d}_{ij}(\mathbf{r})$. This adds complexity in the expressions, but the spirit of the derivation remains unchanged. We now turn to express the vector potential as a function of the $\hat{f}_\lambda$ operators as:

$$\hat{A}(\mathbf{r}) = -i\sum_\lambda\int\frac{d\omega}{\omega}\int d\mathbf{r}'\left[\overline{\overline{G}}_\lambda(\mathbf{r},\mathbf{r}',\omega)\cdot\hat{f}_\lambda(\mathbf{r}',\omega) - \hat{f}^\dagger_\lambda(\mathbf{r}',\omega)\cdot\overline{\overline{G}}^\dagger_\lambda(\mathbf{r},\mathbf{r}',\omega)\right] \tag{s25}$$

Plugging this into the previous Equation and making the aforementioned substitutions leads to

$$\hat{p}\cdot\hat{A} = \frac{-\hbar}{2}\sum_{i,j}\sum_{\lambda}\iiint d\mathbf{r}\,d\mathbf{r}'\,\frac{d\omega}{\omega}\Big[\hat{\sigma}_{ij}\,\mathbf{d}_{ij}(\mathbf{r})\cdot\overline{\overline{G}}_{\lambda}(\mathbf{r},\mathbf{r}',\omega)\cdot\hat{f}_{\lambda}(\mathbf{r}',\omega)+ \\ -\hat{\sigma}_{ij}\,\hat{f}^{\dagger}_{\lambda}(\mathbf{r}',\omega)\cdot\overline{\overline{G}}^{\dagger}_{\lambda}(\mathbf{r},\mathbf{r}',\omega)\cdot\mathbf{d}_{ij}(\mathbf{r}) \\ -\mathbf{d}^{*}_{ij}(\mathbf{r})\cdot\overline{\overline{G}}_{\lambda}(\mathbf{r},\mathbf{r}',\omega)\cdot\hat{f}_{\lambda}(\mathbf{r}',\omega)\,\hat{\sigma}^{\dagger}_{ij} \\ +\hat{f}^{\dagger}_{\lambda}(\mathbf{r}',\omega)\cdot\overline{\overline{G}}^{\dagger}_{\lambda}(\mathbf{r},\mathbf{r}',\omega)\cdot\mathbf{d}^{*}_{ij}(\mathbf{r})\,\hat{\sigma}^{\dagger}_{ij}\Big] \tag{s26}$$

In order to obtain direct interaction terms between the different electronic transitions, it is necessary to obtain the dynamics of the $\hat{f}$ operators in terms of the electronic transition operators. The time evolution of any operator in the Heisenberg picture is given by $\dot{\hat{O}}(t) = -\frac{i}{\hbar}[\hat{O}(t),\hat{H}]$. In particular, it is useful to split the complete hamiltonian into the field + electronic system hamiltonian, $\hat{H}_s$, and the interaction hamiltonian $\hat{H}_I = e\,\hat{p}\cdot\hat{A}/m$. This way the evolution of $\hat{O}$ is given by:

$$\dot{\hat{O}}(t) = -\frac{i}{\hbar}[\hat{O}(t),\hat{H}_s]+i\frac{e}{2m}\sum_{i,j}\sum_{\lambda}\iiint d\mathbf{r}\,d\mathbf{r}'\,\frac{d\omega}{\omega}\times \\ \Bigg[[\hat{O},\hat{\sigma}_{ij}]\Big(\mathbf{d}_{ij}(\mathbf{r})\cdot\overline{\overline{G}}_{\lambda}(\mathbf{r},\mathbf{r}',\omega)\cdot\hat{f}_{\lambda}(\mathbf{r}',\omega)-\hat{f}^{\dagger}_{\lambda}(\mathbf{r}'.\omega)\cdot\overline{\overline{G}}^{\dagger}_{\lambda}(\mathbf{r},\mathbf{r}',\omega)\cdot\mathbf{d}_{ij}(\mathbf{r})\Big) \\ -\Big(\mathbf{d}^{*}_{ij}(\mathbf{r})\cdot\overline{\overline{G}}_{\lambda}(\mathbf{r},\mathbf{r}',\omega)\cdot\hat{f}_{\lambda}(\mathbf{r}',\omega)-\hat{f}^{\dagger}_{\lambda}(\mathbf{r}'.\omega)\cdot\overline{\overline{G}}^{\dagger}_{\lambda}(\mathbf{r},\mathbf{r}',\omega)\cdot\mathbf{d}^{*}_{ij}(\mathbf{r})\Big)[\hat{O},\hat{\sigma}^{\dagger}_{ij}]\Bigg] \tag{s27}$$

In particular, the time evolution of the fundamental field operators $\hat{f}_{\lambda}$ reads:

$$\dot{\hat{f}}_{\lambda'}(\mathbf{r}',\omega',t) = -\frac{i}{\hbar}\left(\sum_{\lambda}\iint dr d\omega\,\hbar\omega\,\left[\hat{f}_{\lambda'}(\mathbf{r}',\omega',t),\hat{f}^{\dagger}_{\lambda}(\mathbf{r},\omega,t)\right]\hat{f}_{\lambda}(\mathbf{r},\omega,t)+\frac{e}{m}\left[\hat{f}_{\lambda'}(\mathbf{r}',\omega',t),\hat{p}\cdot\hat{A}\right]\right) \\ = -i\omega'\hat{f}_{\lambda'}(\mathbf{r}',\omega',t)+\frac{ie}{2m\omega'}\sum_{i,j}\int d\mathbf{r}\left[\overline{\overline{G}}^{\dagger}_{\lambda'}(\mathbf{r},\mathbf{r}',\omega')\cdot\mathbf{d}^{*}_{ij}(\mathbf{r})\,\sigma^{\dagger}_{ij}-\sigma_{ij}\,\overline{\overline{G}}^{\dagger}_{\lambda'}(\mathbf{r},\mathbf{r}',\omega')\cdot\mathbf{d}_{ij}(\mathbf{r})\right]$$

Where we have used that $\left[\hat{f}_{\lambda'}(\mathbf{r}',\omega',t),\hat{f}^{\dagger}_{\lambda}(\mathbf{r},\omega,t)\right] = \delta_{\lambda,\lambda'}\delta(\omega-\omega')\delta(\mathbf{r}-\mathbf{r}')$ and Equation (s26). Solving this equation formally we find

$$\hat{f}_{\lambda'}(\mathbf{r}',\omega,t) = \hat{f}^{\text{free}}_{\lambda'}(\mathbf{r}',\omega,t)-i\frac{e}{2m\omega}\sum_{k,l}\times \\ \left\{\iint_0^t dt'\,d\mathbf{r}''\left[\sigma_{kl}(t')\overline{\overline{G}}^{\dagger}_{\lambda'}(\mathbf{r}'',\mathbf{r}',\omega)\cdot\mathbf{d}_{kl}(\mathbf{r}'')-\overline{\overline{G}}^{\dagger}_{\lambda'}(\mathbf{r}'',\mathbf{r}',\omega)\cdot\mathbf{d}^{*}_{kl}(\mathbf{r}'')\,\sigma^{\dagger}_{kl}(t')\right]e^{-i\omega(t-t')}\right\}$$

plugging this back into Equation (s27), expanding the product and making use of the identity $\sum_\lambda \int d\mathbf{s}\overline{\overline{G}}_\lambda(\mathbf{r},\mathbf{s},\omega)\cdot \overline{\overline{G}}^\dagger_\lambda(\mathbf{r}'',\mathbf{s},\omega) = \frac{\hbar\mu_0}{\pi}\omega^2\mathrm{Im}(\overline{\overline{G}}(\mathbf{r},\mathbf{r}'',\omega))$ [3], the equation of motion for $\hat{O}$ reads

$$\dot{\hat{O}}(t) = -\frac{i}{\hbar}[\hat{O}(t),\hat{H}_s] + i\frac{e^2}{4m^2}\frac{\hbar\mu_0}{\pi}\sum_{\substack{i,j\\k,l}}\int_0^t dt' \iiint d\mathbf{r}\, d\mathbf{r}''\, d\omega \times$$

$$\Big[- i[\hat{O},\sigma_{ij}]\sigma_{kl}(t')\mathbf{d}_{ij}(\mathbf{r})\cdot \mathrm{Im}\left\{\overline{\overline{G}}(\mathbf{r},\mathbf{r}'',\omega)\right\}\cdot \mathbf{d}_{kl}(\mathbf{r}'')e^{-i\omega(t-t')}$$
$$+ i[\hat{O},\sigma_{ij}]\sigma^\dagger_{kl}(t')\mathbf{d}_{ij}(\mathbf{r})\cdot \mathrm{Im}\left\{\overline{\overline{G}}(\mathbf{r},\mathbf{r}'',\omega)\right\}\cdot \mathbf{d}^*_{kl}(\mathbf{r}'')\, e^{-i\omega(t-t')}$$
$$- i[\hat{O},\sigma_{ij}]\sigma^\dagger_{kl}(t')\, \mathbf{d}^*_{kl}(\mathbf{r}'')\cdot \mathrm{Im}\left\{\overline{\overline{G}}(\mathbf{r}'',\mathbf{r},\omega)\right\}\cdot \mathbf{d}_{ij}(\mathbf{r})e^{i\omega(t-t')}$$
$$+ i[\hat{O},\sigma_{ij}]\sigma_{kl}(t')\mathbf{d}_{kl}(\mathbf{r}'')\cdot \mathrm{Im}\left\{\overline{\overline{G}}(\mathbf{r}'',\mathbf{r},\omega)\right\}\cdot \mathbf{d}_{ij}(\mathbf{r})e^{i\omega(t-t')}$$
$$+ i\mathbf{d}^*_{ij}(\mathbf{r})\cdot \mathrm{Im}\left\{\overline{\overline{G}}(\mathbf{r},\mathbf{r}'',\omega)\right\}\cdot \mathbf{d}_{kl}(\mathbf{r}'')\sigma_{kl}(t')[\hat{O},\sigma^\dagger_{ij}]e^{-i\omega(t-t')}$$
$$- i\mathbf{d}^*_{ij}(\mathbf{r})\cdot \mathrm{Im}\left\{\overline{\overline{G}}(\mathbf{r},\mathbf{r}'',\omega)\right\}\cdot \mathbf{d}^*_{kl}(\mathbf{r}'')\,\sigma^\dagger_{kl}(t')[\hat{O},\sigma^\dagger_{ij}]e^{-i\omega(t-t')}$$
$$+ i\mathbf{d}^*_{kl}(\mathbf{r}'')\cdot \mathrm{Im}\left\{\overline{\overline{G}}(\mathbf{r}'',\mathbf{r},\omega)\right\}\cdot \mathbf{d}^*_{ij}(\mathbf{r})\sigma^\dagger_{kl}(t')[\hat{O},\sigma^\dagger_{ij}]e^{i\omega(t-t')}$$
$$- i\mathbf{d}_{kl}(\mathbf{r}'')\mathrm{Im}\left\{\overline{\overline{G}}(\mathbf{r}'',\mathbf{r},\omega)\right\}\cdot \mathbf{d}^*_{ij}(\mathbf{r})\,\sigma_{kl}(t')[\hat{O},\sigma^\dagger_{ij}]e^{i\omega(t-t')}\Big]$$

Now we introduce a coarse-graining Markov approximation, approximating the time dependency of the ladder operators by the natural frequency of the shifted eigenenergies[1]: $\hat{\sigma}_{ij}(t') \approx \tilde{\hat{\sigma}}_{ij}(t)e^{-i\omega_{ij}t'}$. This in turn means that $\tilde{\hat{\sigma}}_{ij}(t) = \hat{\sigma}_{ij}(t)e^{i\omega_{ij}t}$, and therefore $\hat{\sigma}_{ij}(t') \approx \hat{\sigma}_{ij}(t)e^{i\omega_{ij}(t-t')}$. Then we make the substitution $\int dt' e^{i\Omega(t-t')} \to \xi(\Omega)$, where $\xi(x) = \pi\delta(x) + i\mathcal{P}\left(1/x\right)$ by virtue of the Sokhotski-Plemejl theorem, where $\mathcal{P}$ denotes the principal value. These two terms of the integral represent the resonant (the δ function) and the non resonant (the principal value) part of the interaction. In what follows, we assume that the field-mediated interaction between the two electronic transitions is happening through the non-resonant electromagnetic environment, and therefore only consider the principal value contribution. In this situation

1 Solving a Heisemberg equation for the time evolution of these ladder operators one can see that $\omega_{ij} = \omega_j - \omega_i$.

$\xi(-x) = \xi^*(x)$, and the time evolution of $\hat{O}$ reads:

$$\dot{\hat{O}}(t) = -\frac{i}{\hbar}[\hat{O}(t), \hat{H}_s] + i\frac{e^2}{4m^2}\frac{\hbar\mu_0}{\pi}\sum_{\substack{i,j\\k,l}}\iiint d\mathbf{r}\, d\mathbf{r}''\, d\omega \times$$

$$\Big[- i[\hat{O},\sigma_{ij}]\sigma_{kl}\mathbf{d}_{ij}(\mathbf{r})\cdot \mathrm{Im}\left\{\overline{\overline{G}}(\mathbf{r},\mathbf{r}'',\omega)\right\}\cdot \mathbf{d}_{kl}(\mathbf{r}'')\xi(-(\omega-\omega_{kl}))$$
$$+ i[\hat{O},\sigma_{ij}]\sigma_{kl}^\dagger\mathbf{d}_{ij}(\mathbf{r})\cdot \mathrm{Im}\left\{\overline{\overline{G}}(\mathbf{r},\mathbf{r}'',\omega)\right\}\cdot \mathbf{d}_{kl}^*(\mathbf{r}'')\,\xi(-(\omega+\omega_{kl}))$$
$$- i[\hat{O},\sigma_{ij}]\sigma_{kl}^\dagger\,\mathbf{d}_{kl}^*(\mathbf{r}'')\cdot \mathrm{Im}\left\{\overline{\overline{G}}(\mathbf{r}'',\mathbf{r},\omega)\right\}\cdot \mathbf{d}_{ij}(\mathbf{r})\xi(\omega-\omega_{kl})$$
$$+ i[\hat{O},\sigma_{ij}]\sigma_{kl}\mathbf{d}_{kl}(\mathbf{r}'')\cdot \mathrm{Im}\left\{\overline{\overline{G}}(\mathbf{r}'',\mathbf{r},\omega)\right\}\cdot \mathbf{d}_{ij}(\mathbf{r})\xi(\omega+\omega_{kl})$$
$$+ i\mathbf{d}_{ij}^*(\mathbf{r})\cdot \mathrm{Im}\left\{\overline{\overline{G}}(\mathbf{r},\mathbf{r}'',\omega)\right\}\cdot \mathbf{d}_{kl}(\mathbf{r}'')\sigma_{kl}[\hat{O},\sigma_{ij}^\dagger]\xi(-(\omega-\omega_{kl}))$$
$$- i\mathbf{d}_{ij}^*(\mathbf{r})\cdot \mathrm{Im}\left\{\overline{\overline{G}}(\mathbf{r},\mathbf{r}'',\omega)\right\}\cdot \mathbf{d}_{kl}^*(\mathbf{r}'')\,\sigma_{kl}^\dagger[\hat{O},\sigma_{ij}^\dagger]\xi(-(\omega+\omega_{kl}))$$
$$+ i\mathbf{d}_{kl}^*(\mathbf{r}'')\cdot \mathrm{Im}\left\{\overline{\overline{G}}(\mathbf{r}'',\mathbf{r},\omega)\right\}\cdot \mathbf{d}_{ij}^*(\mathbf{r})\sigma_{kl}^\dagger[\hat{O},\sigma_{ij}^\dagger]\xi(\omega-\omega_{kl})$$
$$- i\mathbf{d}_{kl}(\mathbf{r}'')\mathrm{Im}\left\{\overline{\overline{G}}(\mathbf{r}'',\mathbf{r},\omega)\right\}\cdot \mathbf{d}_{ij}^*(\mathbf{r})\,\sigma_{kl}[\hat{O},\sigma_{ij}^\dagger]\xi(\omega+\omega_{kl})\Big]$$

Using several of the exchange symmetries of the indices, namely $d_{ji} = -d_{ij}^*$ and that $\sigma_{kl} = \sigma_{lk}^\dagger$, and $\omega_{lk} = -\omega_{kl}$, allows to rewrite the previous Heisenberg equation in a more compact form

$$\dot{\hat{O}}(t) = -\frac{i}{\hbar}[\hat{O}(t), \hat{H}_s] + \frac{i}{\hbar}\frac{e^2}{2m^2}\frac{\hbar^2\mu_0}{\pi}\sum_{\substack{i,j\\k,l}}\left[[\hat{O},\sigma_{ij}]\sigma_{kl}^\dagger f_{ij,kl} + f_{ij,kl}^*\sigma_{kl}[\hat{O},\sigma_{ij}^\dagger]\right] \tag{s28}$$

where we have defined:

$$f_{ij,kl} = \iint d\mathbf{r}\, d\mathbf{r}'\, \mathbf{d}_{ij}(\mathbf{r})\cdot\left\{\mathcal{P}\int_0^\infty d\omega\left[\frac{\mathrm{Im}\left\{\overline{\overline{G}}(\mathbf{r},\mathbf{r}',\omega)\right\}}{\omega_{kl}+\omega} + \frac{\mathrm{Im}\left\{\overline{\overline{G}}(\mathbf{r},\mathbf{r}',\omega)\right\}}{\omega-\omega_{kl}}\right]\right\}\cdot \mathbf{d}_{kl}^*(\mathbf{r}') \tag{s29}$$

In these coefficients, we have integrals regarding the imaginary part of the system greens function. We can use the kramers-kronig relations to perform these integrals in the following way:

$$\mathcal{P}\int_0^\infty d\omega\,\frac{\mathrm{Im}\left\{\overline{\overline{G}}(\mathbf{s},\mathbf{t},\omega)\right\}}{\omega\pm\omega_0} = \mathcal{P}\int_{-\infty}^\infty d\omega\,\frac{\mathrm{Im}\left\{\overline{\overline{G}}(\mathbf{s},\mathbf{t},\omega)\right\}}{\omega\pm\omega_0} - \mathcal{P}\int_{-\infty}^0 d\omega\,\frac{\mathrm{Im}\left\{\overline{\overline{G}}(\mathbf{s},\mathbf{t},\omega)\right\}}{\omega\pm\omega_0} =$$
$$= \pi\mathrm{Re}\left\{\overline{\overline{G}}(\mathbf{s},\mathbf{t},\mp\omega_0)\right\} - \mathcal{P}\int_0^\infty d\omega\,\frac{\mathrm{Im}\left\{\overline{\overline{G}}(\mathbf{s},\mathbf{t},\omega)\right\}}{\omega\mp\omega_0}$$

Where we have used the following property of the greens function $\overline{\overline{G}}^*(\mathbf{s},\mathbf{t},\omega) = \overline{\overline{G}}(\mathbf{s},\mathbf{t},-\omega^*)$. Taking into account that the Dyadic is a symmetric tensor and that the spatial coordinates can be exchanged [4], together with the relation we have just shown, we can further simplify the constants as

$$f_{ij,kl} = \pi\iint d\mathbf{r}\, d\mathbf{r}'\, \mathbf{d}_{ij}(\mathbf{r})\cdot \mathrm{Re}\left\{\overline{\overline{G}}(\mathbf{r},\mathbf{r}',|\omega_{kl}|)\right\}\cdot \mathbf{d}_{kl}^*(\mathbf{r}').$$

By further manipulating the master equation it can be cast in the following form:

$$\dot{\hat{O}}(t) = -\frac{i}{\hbar}[\hat{O}(t), \hat{H}_s]$$
$$+\frac{i}{\hbar}\frac{e^2}{2m^2}\frac{\hbar^2\mu_0}{\pi}\sum_{\substack{i,j\\k,l}}\left[\frac{f_{ij,kl}+f_{kl,ij}^*}{2}\left[\hat{O},\sigma_{ij}\sigma_{kl}^\dagger\right] + \frac{f_{ij,kl}-f_{kl,ij}^*}{2}\left[\left\{\hat{O},\sigma_{ij}\sigma_{kl}^\dagger\right\} - 2\sigma_{ij}\hat{O}\sigma_{kl}^\dagger\right]\right] \tag{s30}$$

we recognize two terms of different nature: the first one is hermitian, and captures a coherent coupling between the two electronic transitions mediated by the EM fields. The second term has a lindblad-like form, and describes the dissipative coupling between these two transitions. Explicitly writing the coefficients of these two terms:

$$f_{ij,kl} + f^*_{kl,ij} = \pi \iint d\mathbf{r}\, d\mathbf{r}'\, \mathbf{d}_{ij}(\mathbf{r}) \cdot \left[\mathrm{Re}\left\{\overline{\overline{G}}(\mathbf{r},\mathbf{r}',|\omega_{kl}|)\right\} + \mathrm{Re}\left\{\overline{\overline{G}}(\mathbf{r},\mathbf{r}',|\omega_{ij}|)\right\}\right] \cdot \mathbf{d}^*_{kl}(\mathbf{r}'),$$
$$f_{ij,kl} - f^*_{kl,ij} = \pi \iint d\mathbf{r}\, d\mathbf{r}'\, \mathbf{d}_{ij}(\mathbf{r}) \cdot \left[\mathrm{Re}\left\{\overline{\overline{G}}(\mathbf{r},\mathbf{r}',|\omega_{kl}|)\right\} - \mathrm{Re}\left\{\overline{\overline{G}}(\mathbf{r},\mathbf{r}',|\omega_{ij}|)\right\}\right] \cdot \mathbf{d}^*_{kl}(\mathbf{r}'),$$

one sees that if the two electronic transitions involved are resonant or happen in frequencies in which the Green's function varies slowly, then $f_{ij,kl} \approx f^*_{kl,ij}$, and then the dynamics of the field mediated electronic interaction will be described by an effective hamiltonian $\dot{\hat{O}}(t) = -\frac{i}{\hbar}[\hat{O}(t), \hat{H}_{eff}]$ and the hamiltonian is defined as:

$$\hat{H}_{eff} = \hat{H}_s - \sum_{ij} \hbar g^{m-m}_{ij,ij} \hat{c}^\dagger_i \hat{c}_i - \sum_{\substack{i,j \\ k,l \neq i,j}} \hbar g^{m-m}_{ij,kl} \sigma_{ij} \sigma^\dagger_{kl} \tag{s31}$$

$$g^{m-m}_{ij,kl} = \frac{e^2 \hbar \mu_0}{2m^2} \iint d\mathbf{r}\, d\mathbf{r}'\, \mathbf{d}_{ij}(\mathbf{r}) \cdot \mathrm{Re}\left\{\overline{\overline{G}}\left(\mathbf{r},\mathbf{r}',\frac{|\omega_{kl}| + |\omega_{ij}|}{2}\right)\right\} \cdot \mathbf{d}^*_{kl}(\mathbf{r}') \tag{s32}$$

Where we have introduced an effective matter-matter coupling strength $g^{m-m}_{ij,kl}$. Note that in Equation (s31) we distinguish between cases in which $\{kl\} \neq \{ij\}$, and those in which $\{kl\} = \{ij\}$. While the first summation terms describe this coherent coupling between electronic transitions, the second kind renormalizes the energy of the electronic eigenstates with an energy shift given by $\hbar\delta_i = \sum_j g^{m-m}_{ij,ij}$. From the definition of the coupling strength we see that $g^{m-m}_{ij,kl} = g^{m-m*}_{kl,ij}$, and also, since $\mathbf{d}_{ji} = -\mathbf{d}^*_{ij}$, $g^{m-m}_{ji,lk} = g^{m-m*}_{ij,kl}$. The complete hamiltonian then reads:

$$\hat{H}_{\text{eff}} = \sum_\lambda \iint d\mathbf{r}\, d\omega\, \hbar\omega \hat{f}^\dagger_\lambda(\mathbf{r},\omega) \hat{f}_\lambda(\mathbf{r},\omega) + \sum_i (E_i - \hbar\delta_i)\, \hat{c}^\dagger_i \hat{c}_i$$
$$+ \frac{e}{m}\hat{p} \cdot \hat{A}' - \sum_{\substack{i,j \\ k,l \neq i,j}} \hbar\, g^{m-m}_{ij,kl}\, \sigma_{ij} \sigma^\dagger_{kl} \tag{s33}$$

The first two terms in Equation (s33) correspond to the energies of the field and electronic states, while the second line describes the coupling between the field and the electronic states and among electronic transitions. It is important to notice that the interaction of the electronic states with the field is mediated by the resonant frequency part, which we did not account for in the derivation of the last term[2]. On the next section, we will focus on writing the light-matter coupling term of the hamiltonian in a similar fashion as we have done here.

S2.2 Light-matter interaction: Current centered modes

Next, our interest lies in finding how to express the light matter couplings in terms of generalized bosonic operators. For that we revisit the resonant term in the minimal coupling in Equation (s26). Writing:

$$\hat{p} = -i\frac{\hbar}{2} \sum_{i,j} \int d\mathbf{r} \left[\hat{\sigma}_{ij}\, \phi^*_i(\mathbf{r}) \nabla \phi_j(\mathbf{r}) - \hat{\sigma}_{ji}\, \phi_i(\mathbf{r}) \nabla \phi^*_j(\mathbf{r})\right]$$
$$\hat{A}(\mathbf{r}) = -i \sum_\lambda \int \frac{d\omega}{\omega} \int d\mathbf{r}' \left[\overline{\overline{G}}_\lambda(\mathbf{r},\mathbf{r}',\omega) \cdot \hat{f}_\lambda(\mathbf{r}',\omega) - \hat{f}^\dagger_\lambda(\mathbf{r}'.\omega) \cdot \overline{\overline{G}}^\dagger_\lambda(\mathbf{r},\mathbf{r}',\omega)\right]$$

2 See the Sokhotski-Plemejl decomposition made in page 6

The interaction hamiltonian term looks like

$$H_I = \frac{e}{m}\hat{p}\cdot\hat{A} = -\frac{e\hbar}{2m}\sum_{i,j}\sum_{\lambda}\int\frac{d\omega}{\omega}\Bigg\{\hat{\sigma}_{ij}\iint d\mathbf{r}\,d\mathbf{r}'\,\mathbf{d}_{ij}(\mathbf{r})\cdot\overline{\overline{G}}_\lambda(\mathbf{r},\mathbf{r}',\omega)\cdot\hat{f}_\lambda(\mathbf{r}',\omega)$$
$$+\hat{\sigma}_{ji}\iint d\mathbf{r}\,d\mathbf{r}'\,\mathbf{d}_{ji}(\mathbf{r})\cdot\overline{\overline{G}}_\lambda(\mathbf{r},\mathbf{r}',\omega)\cdot\hat{f}_\lambda(\mathbf{r}',\omega)$$
$$+\hat{\sigma}_{ij}\iint d\mathbf{r}\,d\mathbf{r}'\,\hat{f}^\dagger_\lambda(\mathbf{r}',\omega)\cdot\overline{\overline{G}}^\dagger_\lambda(\mathbf{r},\mathbf{r}',\omega)\cdot\mathbf{d}^*_{ji}(\mathbf{r})$$
$$+\hat{\sigma}_{ji}\iint d\mathbf{r}\,d\mathbf{r}'\,\hat{f}^\dagger_\lambda(\mathbf{r}',\omega)\cdot\overline{\overline{G}}^\dagger_\lambda(\mathbf{r},\mathbf{r}',\omega)\cdot\mathbf{d}^*_{ij}(\mathbf{r})\Bigg\} \tag{s34}$$

where to get the last term we have just exchanged the indices and used that $\mathbf{d}_{ji}(\mathbf{r}) = -\mathbf{d}^*_{ij}(\mathbf{r})$. Following the spirit of emitter centerd modes [2, 5] we rewrite the hamiltonian by defining a new set of bosonic operators as

$$H_I = \hbar\sum_{i,j}\int d\omega\left[g_{ij}(\omega)\sigma_{ij}\,\hat{a}_{ij}(\omega) + g_{ji}(\omega)\sigma_{ji}\,\hat{a}_{ji}(\omega) + g^*_{ij}(\omega)\sigma^\dagger_{ij}\,\hat{a}^\dagger_{ij}(\omega) + g^*_{ji}(\omega)\sigma^\dagger_{ji}\,\hat{a}^\dagger_{ji}(\omega)\right] \tag{s35}$$

$$\hat{a}_{ij}(\omega) = \frac{-e\sum_\lambda}{2m\omega g_{ij}(\omega)}\iint d\mathbf{r}\,d\mathbf{r}'\,\mathbf{d}_{ij}(\mathbf{r})\cdot\overline{\overline{G}}_\lambda(\mathbf{r},\mathbf{r}',\omega)\cdot\hat{f}_\lambda(\mathbf{r}',\omega) \tag{s36}$$

$$\hat{a}^\dagger_{ij}(\omega) = \frac{-e\sum_\lambda}{2m\omega g^*_{ij}(\omega)}\iint d\mathbf{r}\,d\mathbf{r}'\,\hat{f}^\dagger_\lambda(\mathbf{r}',\omega)\cdot\overline{\overline{G}}^\dagger_\lambda(\mathbf{r},\mathbf{r}',\omega)\cdot\mathbf{d}^*_{ij}(\mathbf{r}) \tag{s37}$$

From these expressions and the conmutation properties of the $\hat{f}$ operators, it can be shown that $[\hat{a}_{ij},\hat{a}_{kl}] = 0$. By using the conmutation relations of the $\hat{f}$ operators and the identity $\sum_\lambda\int ds\overline{\overline{G}}_\lambda(\mathbf{r},\mathbf{s},\omega)\cdot\overline{\overline{G}}^\dagger_\lambda(\mathbf{r}'',\mathbf{s},\omega) = \frac{\hbar\mu_0}{\pi}\omega^2\mathrm{Im}(\overline{\overline{G}}(\mathbf{r},\mathbf{r}'',\omega))$, the conmutation relation for these new bosonic operators is given by:

$$[\hat{a}_{ij}(\omega),\hat{a}^\dagger_{kl}(\omega')] = \delta(\omega-\omega')\frac{e^2\hbar\mu_0}{4m^2\pi g_{ij}(\omega)g^*_{kl}(\omega)}\iint d\mathbf{r}d\mathbf{r}''\,\mathbf{d}_{ij}(\mathbf{r})\cdot\mathrm{Im}\left\{\overline{\overline{G}}(\mathbf{r},\mathbf{r}'',\omega)\right\}\cdot\mathbf{d}^*_{kl}(\mathbf{r}'')$$

In particular, by imposing $[\hat{a}_{ij},\hat{a}^\dagger_{ij}] \equiv 1$, we find that the coupling between the photon mode and the electronic transition is given by:

$$g_{ij}(\omega) = \frac{e}{2m}\sqrt{\frac{\hbar\mu_0}{\pi}\iint d\mathbf{r}\,d\mathbf{r}'\,\mathbf{d}_{ij}(\mathbf{r})\cdot\mathrm{Im}\left\{\overline{\overline{G}}(\mathbf{r},\mathbf{r}',\omega)\right\}\cdot\mathbf{d}^*_{ij}(\mathbf{r}')} \tag{s38}$$

This is the central result of this section. It shows that the coupling strength between a given electronic transition and an optical mode can be written in a form very similar to the coupling strength between a QE and an optical mode, albeit replacing the puntual dipole moment by an extended current distribution. Using the fact that the greens function is a symmetric tensor, it can be seen that g_{ij} is a real constant, and that $g_{ij} = g_{ji}$. This then allows to rewrite the general conmutator as

$$[\hat{a}_{ij}(\omega),\hat{a}^\dagger_{kl}(\omega')] = \delta(\omega-\omega')\frac{F^{kl}_{ij}}{\sqrt{F^{ij}_{ij}F^{kl}_{kl}}} \tag{s39}$$

$$F^{cd}_{ab} = \iint d\mathbf{r}d\mathbf{r}'\,\mathbf{d}_{ab}(\mathbf{r})\cdot\mathrm{Im}\left\{\overline{\overline{G}}(\mathbf{r},\mathbf{r}',\omega)\right\}\cdot\mathbf{d}^*_{cd}(\mathbf{r}') \tag{s40}$$

Although we will not tackle this problem here, the modes defined in this manner are not orthogonal, since the conmutators for the modes from different transitions are non-zero. Some strategies have been proposed to tackle this problem and generate a set of field operators that do conmute among themselves [2]. We are particularly interested in systems in which the Green's funciton is sharply peaked around a resonant frequency: ω_c. By writting the Greens function as

$$\overline{\overline{G}}(\mathbf{r},\mathbf{r}',\omega) = \overline{\overline{\mathcal{G}}}(\mathbf{r},\mathbf{r}',\omega_c)\delta(\omega-\omega_c), \tag{s41}$$

the interaction hamiltonian in Equation (s35) can be written as a familiar Rabi interaction hamiltonian:

$$H_I = \hbar \sum_{i,j} g_{ij}^{l-m} \left[\sigma_{ij} \left(\hat{a}_{ij} + \hat{a}_{ji}^\dagger \right) + \sigma_{ij}^\dagger \left(\hat{a}_{ji} + \hat{a}_{ij}^\dagger \right) \right] \tag{s42}$$

$$\hat{a}_{ij} = \frac{-e \sum_\lambda}{2m\omega g_{ij}} \iint d\mathbf{r}\, d\mathbf{r'}\, \mathbf{d}_{ij}(\mathbf{r}) \cdot \overline{\overline{\mathcal{G}}}_\lambda(\mathbf{r}, \mathbf{r'}, \omega_c) \cdot \hat{f}_\lambda(\mathbf{r'}, \omega_c) \tag{s43}$$

$$g_{ij}^{l-m} = \frac{e}{2m} \sqrt{\frac{\hbar\mu_0}{\pi} \iint d\mathbf{r}\, d\mathbf{r'}\, \mathbf{d}_{ij}(\mathbf{r}) \cdot \mathrm{Im}\left\{ \overline{\overline{\mathcal{G}}}(\mathbf{r}, \mathbf{r'}, \omega_c) \right\} \cdot \mathbf{d}_{ij}^*(\mathbf{r'})} \tag{s44}$$

Where we have now introduced a light-matter interaction strength denoted by g_{ij}^{l-m}. Finally, the effective hamiltonian of a system that involves general electronic transitions interacting with a single mode field can be written as

$$\begin{aligned} \hat{H}_{\text{eff}} = &\sum_{ij} \hbar\omega_c \hat{a}_{ij}^\dagger \hat{a}_{ij} + \sum_i \tilde{E}_i \hat{c}_i^\dagger \hat{c}_i \\ &+ \hbar \sum_{i,j} g_{ij}^{l-m} \hat{\sigma}_{ij} \left(\hat{a}_{ij} + \hat{a}_{ji}^\dagger \right) \\ &- \sum_{\substack{i,j \\ k,l \neq i,j}} \hbar\, g_{ij,kl}^{m-m}\, \sigma_{ij} \sigma_{kl}^\dagger \end{aligned} \tag{s45}$$

with

$$g_{ij}^{l-m} = \frac{e}{m} \sqrt{\frac{\hbar\mu_0}{\pi} \iint d\mathbf{r}\, d\mathbf{r'}\, \mathbf{d}_{ij}(\mathbf{r}) \cdot \mathrm{Im}\left\{ \overline{\overline{\mathcal{G}}}(\mathbf{r}, \mathbf{r'}, \omega_c) \right\} \cdot \mathbf{d}_{ij}^*(\mathbf{r'})} \tag{s46}$$

$$g_{ij,kl}^{m-m} = \frac{e^2\hbar\mu_0}{2m^2} \iint d\mathbf{r}\, d\mathbf{r'}\, \mathbf{d}_{ij}(\mathbf{r}) \cdot \mathrm{Re}\left\{ \overline{\overline{G}} \left(\mathbf{r}, \mathbf{r'}, \frac{|\omega_{kl}| + |\omega_{ij}|}{2} \right) \right\} \cdot \mathbf{d}_{kl}^*(\mathbf{r'}) \tag{s47}$$

S3 Electronic transitions of interest

One of the strengths of the above hamiltonian is it's applicability to a wide variety of electronic transitions. One could study the coupling of bloch electrons in materials, localised electronic transitions in molecules, or bound electrons in material defects on equal footing, provided one is able of obtaining the initial and final electronic states involved in the transition. In this section, we will apply the above formalism to derive couplings for specific cases: localized dipolar electronic transitions and also free electrons.

S3.1 Localized transitions: dipolar quantum emitters

One of the most relevant applications of this framework is when dealing with localised transitions. In this family we can include transitions inside quantum dots, molecules or defects in crystals. This is due to the fact that regardless of the actual shape of the electronic states taking place in the transition, the scale lengths associated with them is much smaller than the scale in which the EM fields change. As such, one could assume that the Green's function is constant over the span of the electronic wavefunctions of the quantum emitter (QE) and write:

$$\begin{aligned} \mathbf{d}_{ij}^{QE}(r) &\approx \delta(r - r_0) \int d\mathbf{r'} \mathbf{d}_{ij}(r') = \delta(r - r_0) \int d\mathbf{r'} \phi_i^*(r') \nabla \phi_j(r') = \delta(r - r_0) \langle i | \nabla | j \rangle = \\ &= -\delta(r - r_0) \frac{m}{\hbar^2} \langle i | [\hat{H}_e, \hat{r}] | j \rangle = -\delta(r - r_0) \frac{m}{e\hbar} (\omega_j - \omega_i) \langle i | -e\hat{r} | j \rangle = -\delta(r - r_0) \frac{m\omega_{ij}}{e\hbar} \boldsymbol{\mu}_{ij} \end{aligned} \tag{s48}$$

where in the end we have the energy difference between the initial and final state given by $\omega_{ij} = \omega_j - \omega_i$, and the usual transition dipole moment given by $\boldsymbol{\mu}_{ij} = \langle i| - e\hat{r}\,|j\rangle$, where e is the electron charge. To arrive at this expression we have used that, in general, the electronic hamiltonian will be written as $\hat{H}_e = V(r) + \hat{p}^2/2m$ and therefore $[\hat{H}_e, \hat{r}] = -i\hbar\hat{p}/m = -\hbar^2\nabla/m$. Putting this transition current density into Equation (s46), we get the very familiar expression for the coupling between a dipolar QE and an optical mode of a cavity:

$$g_{ij}^{c-QE} = \frac{|\omega_{ij}|}{\hbar}\sqrt{\frac{\hbar\mu_0}{\pi}\boldsymbol{\mu}_{ij}\cdot \mathrm{Im}\left\{\overline{\overline{\mathcal{G}}}(\mathbf{r}_0,\mathbf{r}_0,\omega_c)\right\}\cdot\boldsymbol{\mu}_{ij}^*} \tag{s49}$$

Since these couplings are real, $g_{ij}^{c-QE} = g_{ji}^{c-QE} \equiv g^{c-QE}$. For the case of a single QE, with eigenstates named by $|g\rangle$ and $|e\rangle$, for ground and excited state, the interaction between this QE and some EM environment is given (from Equation (s45)) by:

$$\begin{aligned}\hat{H}_I^{c-QE} =& g^{c-QE}\hbar\left[\hat{\sigma}_{ge}\left(\hat{a}_{ge}+\hat{a}_{eg}^\dagger\right)+\hat{\sigma}_{eg}\left(\hat{a}_{eg}+\hat{a}_{ge}^\dagger\right)\right]\\ \approx& \hbar g^{c-QE}\left[\hat{\sigma}\hat{a}_{eg}^\dagger+\hat{\sigma}^\dagger\hat{a}_{eg}\right]\end{aligned} \tag{s50}$$

$$g^{c-QE} = \frac{|\omega_{QE}|}{\hbar}\sqrt{\frac{\hbar\mu_0}{\pi}\boldsymbol{\mu}\cdot \mathrm{Im}\left\{\overline{\overline{\mathcal{G}}}(\mathbf{r}_0,\mathbf{r}_0,\omega_c)\right\}\cdot\boldsymbol{\mu}^*} \tag{s51}$$

where we have introduced the usual QE ladder operators given by $\hat{\sigma} = |g\rangle\langle e|$, defined the QE transition dipole moment as $\boldsymbol{\mu} \equiv \boldsymbol{\mu}_{ge} = \langle g| - e\hat{r}\,|e\rangle$, and applied the rotating wave approximation to the cavity-QE coupling, which leads to the interaction being written in terms of a single optical mode.

S3.2 Free electrons

We will approximate free electron states as momentum eigenstates in the $\hat{z}$ direction with some lateral distribution: $\phi_k(\mathbf{r}) = g_\perp(\mathbf{r}_\perp)e^{ik_kz}/\sqrt{L}$ where L is the length of a fictitious box used to quantize the electron momenta, and $g_\perp(\mathbf{r}_\perp)$ is a function describing the thin lateral profile of the electron wavepacket. In principle we could say that

$$g_\perp(\mathbf{r}_\perp) = \sqrt{\frac{1}{2\pi\sigma^2}}\exp\left\{-\left(\frac{|\mathbf{r}_\perp-\mathbf{r}_{0,\perp}|}{2\sigma}\right)^2\right\},$$

which in the limit of small beam width behaves as a delta in the lateral direction. For a transition between free states we then have:

$$\mathbf{d}_{kl}(\mathbf{r}) = \phi_k^*\nabla\phi_l = \frac{1}{\sigma}\begin{pmatrix}\frac{(x_0-x)}{2\sigma}\\ \frac{(y_0-y)}{2\sigma}\\ ik_l\sigma\end{pmatrix}\frac{e^{-\frac{|\mathbf{r}_\perp-\mathbf{r}_{0,\perp}|^2}{2\sigma^2}}}{2\pi\sigma^2}\frac{e^{i(k_l-k_k)z}}{L} \tag{s52}$$

$$\overset{\lim_{\sigma\to0}}{\approx} ik_0\frac{\delta^2(\mathbf{r}_\perp-\mathbf{r}_{0,\perp})}{L}e^{i(k_l-k_k)z}\hat{z}, \tag{s53}$$

where we have approximated the free electron states as a punctual distribution in a lateral direction and assumed that the electron's momentum doesn't change much within the interaction, and therefore the initial and final electron momentum are given approximately by the incident electron's central momentum, k_0. Of course this also means that we assume that the electron wavepacket has a well defined central momentum. Putting this into Equation (s46)

$$g_{kl}^{e-c} = \frac{ek_0}{mL}\sqrt{\frac{\hbar\mu_0}{\pi}\iint dz\,dz'\;\mathrm{Im}\left\{\hat{z}\cdot\overline{\overline{\mathcal{G}}}([\mathbf{r}_{\perp,0},z],[\mathbf{r}_{\perp,0},z'],\omega_c)\cdot\hat{z}\right\}e^{i(k_l-k_k)(z-z')}} \tag{s54}$$

For an electron interacting with an arbitrary system, initially in the ground state, the first order interaction term will give a probability for the electron to loose a certain amount of momentum proportional to $(g_{kl}^{e-c})^2$. It is then nice to check that the classical EELS probability[6] indeed has the form $(g_{kl}^{e-c})^2$ if one makes

the change $k_l - k_k \to \omega/v_0$, with v_0 being the initial velocity of the electron. We will later see that this substitution is just an implication of energy conservation in the photon exchange between the optical modes and the electron. The fact that we have related the coupling strength to the classical EM Green's function through MQED allows to apply this strategy to arbitrary optical modes and we have the certainty that the normalization of the bosonic operators will be the right one by construction, which implies that this coupling strength expressions are physical.

Also note that the coupling strength above only depends on the momentum of the electron through the momentum change over the interaction. By writing $k_k \equiv k$ and $q \equiv k_l - k_k$ one can see that the above coupling strength can be parametrized just as g_q^{e-c}. The same applies to the optical modes defined in Equation (s43), albeit with the peculiarity that since the cavity's Green function is purely imaginary at the resonance frequency (See Section S4), then one can show from Equation (s43) that $\hat{a}_q = -\hat{a}_{-q}$. With this, the interaction term between a free electron and an optical mode is given by

$$\begin{aligned}\hat{H}_I^{e-c} &= \hbar \sum_q g_q^{e-c} \hat{b}_q \left(\hat{a}_{-q}^\dagger - \hat{a}_{-q}\right) \\ &\approx \hbar \sum_q g_q^{e-c} \hat{b}_q \left(\hat{a}_{-q_0}^\dagger - \hat{a}_{-q_0}\right) \mathrm{sign}(q) \qquad \text{(s55)}\end{aligned}$$

$$\hat{a}_q = \frac{-ik_0 e \sum_\lambda}{2m\omega L g_q} \iint dz\, d\mathbf{r}'\, {}^{iqz} \hat{z} \cdot \overline{\overline{\mathcal{G}}}_\lambda([\mathbf{r}_{\perp,0}, z], \mathbf{r}', \omega_c) \cdot \hat{f}_\lambda(\mathbf{r}', \omega_c) \qquad \text{(s56)}$$

$$g_q^{e-c} = \frac{ek_0}{mL} \sqrt{\frac{\hbar\mu_0}{\pi} \iint dz\, dz'\ \mathrm{Im}\left\{\hat{z} \cdot \overline{\overline{\mathcal{G}}}([\mathbf{r}_{\perp,0}, z], [\mathbf{r}_{\perp,0}, z'], \omega_c) \cdot \hat{z}\right\} e^{iq(z-z')}} \qquad \text{(s57)}$$

Where we have approximated that the optical modes that the electron will interact with are given by those that exchange momentum $q_0 = \omega_c/v_0$. Upon studying the interaction of an electron with an isolated optical mode and enforcing energy conservation, this assertion can be seen to be correct. When considering the interaction of an electron with a polaritonic system, the energy exchanges will differ from ω_c, but since optical plamonic modes are highly spatially confined, their reciprocal space content will be very wide momenta distribution, and therefore, the error included by naming all the optical modes as $\hat{a}_{-q_0}$ is negligible.

In passing, note that the coupling strength above shows that a simple matching condition between the optical modes and the momentum exchange allows to maximize the coupling strength: for an electron interacting with an optical mode of frequency ω_c, energy conservation imposes $q = \omega_c/v_0$. The coupling strength above involves a fourier transform of the associated field profiles, and therefore, for constructive interference one would want that the spatial frequency of the optical modes matches that of $\exp(iqz)$. If we consider a guided mode inside a waveguide, with guided momentum $k = k_0 n_{eff}$, where n_{eff} is the effective refractive index of the guided mode, then we see that by making $k = q = \omega_c/v_0 \to v_0 = c/n_{eff}$, will lead to enhanced interaction strength. This amounts to matching the phase velocity of the optical mode in the waveguide to that of the momentum exchange of the free electron, just as done in [7].

S3.3 Bound electron - free electron interaction

From Equation (s45), and having particularized the current densities for transitions in dipolar QE and free electrons, the hamiltonian describing the free-electron bound electron interaction is given by:

$$\hat{H}_I^{e-QE} = -2\hbar \sum_{kl} \left[g_{ge,lk}^{e-QE}\, \hat{\sigma} + (g_{ge,kl}^{e-QE})^*\, \hat{\sigma}^\dagger\right] \sigma_{kl},$$

where we have introduced the ladder operators of the QE given by $\hat{sigma} = |g\rangle\langle e|$, and explicit expressions for both couplings in the last term are:

$$g^{e-QE}_{ge,lk} = ik_0 \frac{e^2\hbar\mu_0}{2m^2L}\frac{m\omega_{ge}}{e\hbar}\int dz'\,\boldsymbol{\mu}_{ge}\cdot\mathrm{Re}\left\{\overline{\overline{G}}\left(\mathbf{r}_{QE},[\mathbf{r}_{0,\perp},z'],|\omega_{ge}|\right)\right\}\cdot\hat{z}e^{i(k_l-k_k)z'},$$
$$g^{e-QE}_{ge,kl} = ik_0 \frac{e^2\hbar\mu_0}{2m^2L}\frac{m\omega_{ge}}{e\hbar}\int dz'\,\boldsymbol{\mu}_{ge}\cdot\mathrm{Re}\left\{\overline{\overline{G}}\left(\mathbf{r}_{QE},[\mathbf{r}_{0,\perp},z'],|\omega_{ge}|\right)\right\}\cdot\hat{z}e^{-i(k_l-k_k)z'},$$

Defining $k_k = k$ and $k_l = k_k + q$, we see that the two couplings become independent of k

$$g^{e-QE}_{q} \equiv 2g^{e-QE}_{ge,lk} = ik_0 \frac{e^2\hbar\mu_0}{m^2L}\frac{m\omega_{ge}}{e\hbar}\int dz'\,\boldsymbol{\mu}_{ge}\cdot\mathrm{Re}\left\{\overline{\overline{G}}\left(\mathbf{r}_{QE},[\mathbf{r}_{0,\perp},z'],|\omega_{ge}|\right)\right\}\cdot\hat{z}e^{iqz'},$$
$$2g^{e-QE}_{ge,kl} \equiv g^{e-QE}_{-q} = -(g^{e-QE}_{q})^*,$$

which allows to parametrize the interaction hamiltonian entirely in terms of the momentum exchange between the free electron and the QE: q. With this, the interaction hamiltonian and coupling strengths read

$$\hat{H}^{e-QE}_I = -\hbar\sum_q g^{e-QE}_q\left[\hat{\sigma}-\hat{\sigma}^\dagger\right]\hat{b}_q, \tag{s58}$$
$$g^{e-QE}_q = ik_0\frac{e\mu_0\omega_{QE}}{mL}\int dz'\,\boldsymbol{\mu}\cdot\mathrm{Re}\left\{\overline{\overline{G}}\left(\mathbf{r}_{QE},[\mathbf{r}_{0,\perp},z'],|\omega_{QE}|\right)\right\}\cdot\hat{z}e^{iqz'},$$

Notice that the coupling between the QE and the passing electron depends on the real part of the greens function. In our study, the QE and cavity will be in resonance. At the resonance frequency the GF of the NP becomes purely imaginary, and therefore the coupling between the QE and the electron will be mediated by the vacuum GF [4]. In the near field, it reads:

$$\overline{\overline{G_0}}(\mathbf{r},\mathbf{r}') \overset{NF}{\approx} \frac{e^{ikR}}{4\pi R}\frac{1}{k^2R^2}\left[-\overline{\overline{I}}+3\frac{\mathbf{RR}}{R^2}\right].$$

where $\mathbf{R} = \mathbf{r}-\mathbf{r}'$, $R = |\mathbf{R}|$ and $\mathbf{RR}$ denotes the outer product. To calculate $\overline{\overline{G}}(\mathbf{r}_{QE},[\mathbf{r}_\perp,z],\Omega)$, we have $\mathbf{r}_{QE} = [x_{QE},0,z_{QE}]$ $\mathbf{r}_e = [x_{QE}+b_{e-QE},0,z]$, so $\mathbf{R} = \mathbf{r}_{QE}-\mathbf{r}_e = -[b_{e-QE},0,z-z_{QE}]$. If we define $\hat{u} = \mathbf{R}/R$ we then have

$$\boldsymbol{\mu}\cdot\overline{\overline{G}}_0(\mathbf{r}_{QE},[\mathbf{r}_\perp,z])\cdot\hat{z} = \frac{e^{ik\sqrt{b^2_{e-QE}+(z-z_{QE})^2}}}{4\pi\sqrt{b^2_{e-QE}+(z-z_{QE})^2}}\frac{-\boldsymbol{\mu}\cdot\hat{z}+3(\boldsymbol{\mu}\cdot\hat{u})(\hat{u}\cdot\hat{z})}{k^2\left[b^2_{e-QE}+(z-z_{QE})^2\right]}$$
$$\overset{QS}{\approx}\frac{-(\boldsymbol{\mu}\cdot\hat{z})}{4\pi k^2\left[b^2_{e-QE}+(z-z_{QE})^2\right]^{3/2}}+\frac{3(\boldsymbol{\mu}\cdot\hat{x})b_{e-QE}(z-z_{QE})}{4\pi k^2\left[b^2_{e-QE}+(z-z_{QE})^2\right]^{5/2}}+\frac{3(\boldsymbol{\mu}\cdot\hat{z})(z-z_{QE})^2}{4\pi k^2\left[b^2_{e-QE}+(z-z_{QE})^2\right]^{5/2}},$$

Where we have used the quasi-static (QS) limit. So the coupling between the free electron and the 2 level system is given by

$$g^{e-QE}_q = ik_0\frac{e\mu_0\omega_{QE}}{4\pi mLk^2}\frac{e^{iqz_{QE}}\boldsymbol{\mu}}{|b_{e-QE}|^2}\cdot\left\{\left[2I_2(qb_{e-QE})-I_0(qb_{e-QE})\right]\hat{z}+\left[3\,\mathrm{sign}(b_{e-QE}q)I_1(qb_{e-QE})\right]\hat{x}\right\},$$

where we have defined the integrals

$$I_n(\phi) = \int_{-\infty}^{\infty} dz\frac{z^n}{\left[1+z^2\right]^{5/2}}e^{i|\phi|z} \tag{s59}$$

which evaluate to

$$I_0(\phi) = \frac{2}{3}|\phi|^2 K_2(|\phi|), \tag{s60}$$
$$I_1(\phi) = \frac{2i}{3}|\phi|^2 K_1(|\phi|), \tag{s61}$$
$$I_2(\phi) = \frac{2}{3}\left[|\phi|K_1(|\phi|)-|\phi|^2K_0(|\phi|)\right], \tag{s62}$$
$$I_0(\phi)-2I_2(\phi) = 2|\phi|^2K_0(|\phi|).$$

where in the last line we have used the recurrence relation [8] of modified bessel functions to rewrite the expression in a more compact form. From this, the interaction strength between the bound and free electron can be written as:

$$g_q^{e-QE} = -\frac{e\,k_0\,|q|^2\,e^{iqz_{QE}}}{2\pi m L\epsilon_0\omega_{QE}}\boldsymbol{\mu}\cdot\begin{pmatrix}\mathrm{sign}(qb_{e-QE})K_1(|qb_{e-QE}|)\\ 0\\ iK_0(|qb_{e-QE}|)\end{pmatrix}. \tag{s63}$$

Comparing this to a previous result [9] studying the interaction of free and bound electrons, we see that the expression agree, while our expression puts emphasis on the fact that the phase accumulated in the electron interaction with the QE will be different for a situation of energy loss and energy gain.

S4 Quasi-static Greens Function of a Sphere

Here, we aim at obtaining a closed expression for the $\mathcal{G}(\mathbf{r},\mathbf{r}',\omega_c)$ tensor in the Green's function decomposition for the optical mode supported by a spherical cavity:

$$\mathbf{G}(\mathbf{r},\mathbf{r}',\omega) = \mathbf{G}_0(\mathbf{r},\mathbf{r}',\omega) + \mathcal{G}(\mathbf{r},\mathbf{r}')\mathcal{L}(\omega)$$

where $\mathbf{G}_0(\mathbf{r},\mathbf{r}',\omega)$ is the vacuum green's function and $\mathcal{L}(\omega)$ contains the frequency response of the sphere's contribution to the total GF. To do this we solve Poisson equation in the geometry presented in Figure S1 with the charge distribution of an electric dipole:

$$\rho(\mathbf{r}',\omega) = -\mu\cdot\nabla\delta(\mathbf{r}'-\mathbf{r}_\mu)$$

We use that for $r' > r$, we can expand

$$\frac{1}{|\mathbf{r}-\mathbf{r}'|} = \sum_{n=0}^{\infty}\sum_{m=-n}^{n}\frac{4\pi}{2n+1}Y_n^{m*}(\theta',\phi')Y_n^m(\theta,\phi)\frac{r^n}{r'^{n+1}}$$

Which allows to express the potential created by the dipole as

$$\phi_{inc}(\mathbf{r},\omega) = \frac{1}{4\pi\epsilon_0}\int \mathrm{d}\mathbf{r}'\frac{\rho(\mathbf{r}',\omega)}{|\mathbf{r}-\mathbf{r}'|} = \frac{1}{4\pi\epsilon_0}\sum_{n=0}^{\infty}\sum_{m=-n}^{n}\frac{4\pi}{2n+1}I_n^m Y_n^m(\theta,\phi)r^n$$

with

$$I_n^m = \int \mathrm{d}\mathbf{r}'\frac{\rho(\mathbf{r}',\omega)}{r'^{n+1}}Y_n^{m*}(\theta',\phi') = -\nabla'\left(\frac{Y_n^{m*}(\theta',\phi')}{r'^{n+1}}\right)\cdot\boldsymbol{\mu}$$

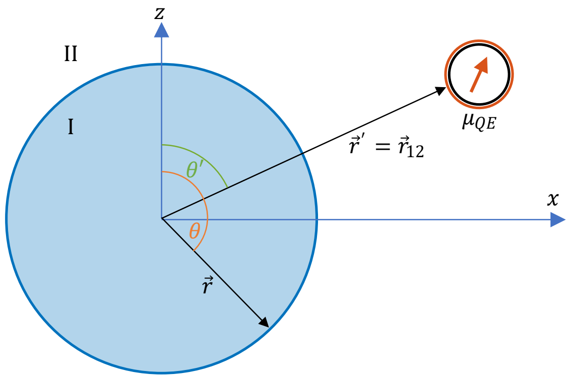

Fig. S1: Sketch of the system under consideration.

Now we can solve the scattering problem by expanding the potential on the different regions and matching spherical harmonics:

$$\begin{aligned}\phi_{I}{}_n^m(\mathbf{r},\omega) &= \phi_{inc}{}_n^m(\mathbf{r},\omega) + \phi_{sc,I}{}_n^m(\mathbf{r},\omega) = \frac{1}{\epsilon_0}\frac{1}{2n+1} I_n^m Y_n^m(\theta,\phi) r^n + A_n^m \frac{Y_n^m(\theta,\phi)}{r^{n+1}} \\ \phi_{II}{}_n^m(\mathbf{r},\omega) &= \phi_{sc,II}{}_n^m(\mathbf{r},\omega) = B_n^m Y_n^m(\theta,\phi) r^n\end{aligned}$$

Imposing continuity of the potential and the normal component of the displacement field we find A_n^m, and hence the scattered field

$$\phi_{sc,I}{}_n^m(\mathbf{r},\omega) = -\left[\frac{1}{\epsilon_0}\frac{R^{2n+1}}{2n+1} I_n^m \frac{\epsilon_{sph}(\omega)-1}{\epsilon_{sph}(\omega)+\frac{n+1}{n}}\right]\frac{Y_n^m(\theta,\phi)}{r^{n+1}}$$

Note that all the frequency dependence is contained within the permittivity of the sphere. If we assume a Drude model for the sphere $\epsilon_{sph} = 1 - \omega_p^2/(\omega(\omega+i\gamma))$, then we can rewrite the permittivity quotient as

$$\frac{\epsilon_{sph}(\omega)-1}{\epsilon_{sph}(\omega)+\frac{n+1}{n}} = \frac{-\frac{\omega_p^2}{\omega(\omega+i\gamma)}}{\frac{2n+1}{n} - \frac{\omega_p^2}{\omega(\omega+i\gamma)}} = \frac{-\omega_{sp}^2}{\omega(\omega+i\gamma)-\omega_{sp}^2}$$

where we have defined $\omega_c = \omega_{sp} = \sqrt{n/(2n+1)}\,\omega_p$, the generalized Frölich poles. The above quotient has poles for $\omega = \pm\sqrt{\omega_{sp}^2 - (\gamma/2)^2} - i\gamma/2$, which in the limit of small losses tends to ω_{sp}. This means that within the good resonator approximation, the above quotient will be only non-zero for a small region around ω_{sp}. Expressing $\omega = \omega_{sp} + \delta$, and expanding for small δ values we find:

$$\frac{\epsilon_{sph}(\omega)-1}{\epsilon_{sph}(\omega)+\frac{n+1}{n}} = \frac{-\omega_{sp}^2}{(\omega_{sp}+\delta)^2 + i\gamma(\omega_{sp}+\delta) - \omega_{sp}^2} \approx -\frac{1}{2}\frac{\omega_{sp}}{\omega - \omega_{sp} + i\frac{\gamma}{2}} = \frac{-\omega_{sp}}{2}\mathcal{L}(\omega,\omega_{sp},\gamma)$$

Which gives a lorentzian lineshape around every frölich pole, with $\mathcal{L}(\omega,\omega_{sp},\gamma) = (\omega - \omega_{sp} + i\gamma/2)^{-1}$. The scattered potential and corresponding field are then

$$\begin{aligned}\phi_{sc,I}{}_n^m(\mathbf{r},\omega) &= \frac{1}{2\epsilon_0}\frac{R^{2n+1}}{2n+1}\omega_{sp}\mathcal{L}(\omega,\omega_{sp},\gamma)\frac{Y_n^m(\theta,\phi)}{r^{n+1}}\nabla'\left(\frac{Y_n^{m*}(\theta',\phi')}{r'^{n+1}}\right)\cdot\boldsymbol{\mu} \\ \mathbf{E}_{sc,I}{}_n^m(\mathbf{r},\omega) &= -\frac{1}{2\epsilon_0}\frac{R^{2n+1}}{2n+1}\omega_{sp}\mathcal{L}(\omega,\omega_{sp},\gamma)\,\nabla\left(\frac{Y_n^m(\theta,\phi)}{r^{n+1}}\right)\nabla'\left(\frac{Y_n^{m*}(\theta',\phi')}{r'^{n+1}}\right)\cdot\boldsymbol{\mu}\end{aligned}$$

By definition the Dyadic Green's function fulfil

$$\mathbf{E}_\mu = \frac{\omega^2}{c^2\epsilon_0}\mathbf{G}(\mathbf{r},\mathbf{r}',\omega))\cdot\boldsymbol{\mu}$$

and therefore the contributions of all the different spherical harmonics to the greens functions are:

$$\mathbf{G}_n^m(\mathbf{r},\mathbf{r}',\omega) = -\frac{c^2}{\omega^2}\frac{R^{2n+1}}{2(2n+1)}\omega_{sp}\mathcal{L}(\omega,\omega_{sp},\gamma)\nabla\left(\frac{Y_n^m(\theta,\phi)}{r^{n+1}}\right)\otimes\nabla'\left(\frac{Y_n^{m*}(\theta',\phi')}{r'^{n+1}}\right)$$

In particular we are interested in the contributions given by the dipolar mode of the nanoparticle, corresponding to $l=1$. Setting $l=1$ inmediately gives three possible values for m, which gives the expected three-fold degeneracy of the dipolar mode. Instead of using the complex formulation, we choose to work with the real-valued spherical harmonics, given by

$$\begin{aligned}\mathcal{Y}_x &= \frac{1}{\sqrt{2}}\left[Y_1^{-1} - Y_1^1\right] = \frac{1}{2}\sqrt{\frac{\pi}{3}}\frac{x}{r} \\ \mathcal{Y}_y &= \frac{1i}{\sqrt{2}}\left[Y_1^{-1} + Y_1^1\right] = \frac{1}{2}\sqrt{\frac{\pi}{3}}\frac{y}{r} \\ \mathcal{Y}_z &= Y_1^0 = \frac{1}{2}\sqrt{\frac{\pi}{3}}\frac{z}{r}\end{aligned}$$

So the greens functions can be expressed as

$$\mathbf{G}_{x,y,z}(\mathbf{r},\mathbf{r}',\omega) = -\frac{c^2}{\omega^2}\frac{\pi R^3}{72}\,\omega_{sp}\mathcal{L}(\omega,\omega_{sp},\gamma)\nabla\left(\frac{x,y,z}{r^3}\right)\otimes\nabla'\left(\frac{x',y',z'}{r'^3}\right)$$

To express this dyadic in the form of Equation (s41), it is enough to note that in the limit of vanishing absorption $\lim_{\gamma\to 0}\mathcal{L}(\omega,\omega_{sp},\gamma) = -i\pi\delta(\omega-\omega_{sp})$, and that $\mathcal{L}(\omega_{sp},\omega_{sp},\gamma) = -2i/\gamma$.

$$\mathbf{G}_{x,y,z}(\mathbf{r},\mathbf{r}',\omega) = \mathcal{G}_{x,y,z}(\mathbf{r},\mathbf{r}',\omega_{sp})\delta(\omega-\omega_{sp}), \tag{s64}$$

$$\mathcal{G}_{x,y,z}(\mathbf{r},\mathbf{r}',\omega_{sp}) = -\frac{\gamma\pi}{2}\frac{c^2}{\omega_{sp}^2}\frac{\pi R^3}{72}\,\omega_{sp}\mathcal{L}(\omega_{sp},\omega_{sp},\gamma)\nabla\left(\frac{x,y,z}{r^3}\right)\otimes\nabla'\left(\frac{x',y',z'}{r'^3}\right). \tag{s65}$$

Finally, noting that $\partial_i(x_j/r^3) = (r^2\delta_{i,j} - 3x_i x_j)/r^5$, the entries of the Greens function of the three degenerated dipolar cavity modes (denoted by x_k) can be written as:

$$\begin{aligned}[\mathcal{G}_{x_k}]_{ij}(\mathbf{r},\mathbf{r}',\omega_{sp}) &= i\pi\frac{c^2}{\omega_{sp}}\frac{\pi R^3}{72}\,\partial_i\left(\frac{x_k}{r^3}\right)\otimes\partial'_j\left(\frac{x'_k}{r'^3}\right)\\ &= i\pi\frac{c^2}{\omega_{sp}}\frac{\pi R^3}{72}\frac{(r^2\delta_{i,k}-3x_i x_k)(r'^2\delta_{j,k}-3x'_j x'_k)}{r^5 r'^5},\end{aligned} \tag{s66}$$

S4.1 Evaluation of QE-cavity coupling

By using Equation (s51), and the Green's function of a sphere from above, the coupling between a QE of dipole moment $\boldsymbol{\mu}$, when the dipole is placed on the $\hat{x}$ axis at a distance b_{c-QE} from the nanoparticle is given by:

$$g_x^{c-QE} = \frac{|\omega_{QE}|}{3}|\boldsymbol{\mu}\cdot\hat{x}|\sqrt{\frac{\pi}{2}\left(\frac{R}{b_{c-QE}}\right)^3\frac{1}{\hbar\omega_{sp}}\frac{1}{\epsilon_0\, b_{c-QE}^3}} \tag{s67}$$

$$g_y^{c-QE} = \frac{|\omega_{QE}|}{6}|\boldsymbol{\mu}\cdot\hat{y}|\sqrt{\frac{\pi}{2}\left(\frac{R}{b_{c-QE}}\right)^3\frac{1}{\hbar\omega_{sp}}\frac{1}{\epsilon_0\, b_{c-QE}^3}} \tag{s68}$$

$$g_z^{c-QE} = \frac{|\omega_{QE}|}{6}|\boldsymbol{\mu}\cdot\hat{z}|\sqrt{\frac{\pi}{2}\left(\frac{R}{b_{c-QE}}\right)^3\frac{1}{\hbar\omega_{sp}}\frac{1}{\epsilon_0\, b_{c-QE}^3}} \tag{s69}$$

which shows that for a dipole oriented along one of the coordinate axis, it only couples to one of the three degenerated dipolar modes of the cavity.

S4.2 Evaluation of free electron - cavity coupling

In the same way, using Equation (s57) and the Green's function of a spherical nanoparticle we find that an electron that passes a distance $\mathbf{r}_{\perp,0} = b_{e-c}\hat{x}$ of the cavity has a coupling to the different dipolar modes given by:

$$\begin{aligned} g_{q,x}^{e-c} &= \frac{ek_0}{mL}\sqrt{\frac{\hbar}{\epsilon_0\omega_{sp}}\frac{\pi R^3}{8}\frac{1}{|b_{e-c}|^4}I_1(qb_{e-c})I_1^*(qb_{e-c})}\\ g_{q,y}^{e-c} &= 0\\ g_{q,z}^{e-c} &= \frac{ek_0}{3mL}\sqrt{\frac{\hbar}{\epsilon_0\omega_{sp}}\frac{\pi R^3}{8}\frac{1}{|b_{e-c}|^4}\left[I_0(qb_{e-c})-2I_2(qb_{e-c})\right]\left[I_0(qb_{e-c})-2I_2(qb_{e-c})\right]^*}\end{aligned}$$

where we have used the integrals defined in Equation (s59). From this, the interaction strength between the passing electron and the dipolar modes of the cavity is given by

$$g_{q,x}^{e-c} = \frac{e\hbar k_0}{3mL}\,|q|^2\,K_1(|qb_{e-c}|)\sqrt{\frac{1}{\epsilon_0\hbar\omega_{sp}}\frac{\pi R^3}{2}} \tag{s70}$$

$$g_{q,y}^{e-c} = 0 \tag{s71}$$

$$g_{q,z}^{e-c} = \frac{e\hbar k_0}{3mL}\,|q|^2\,K_0(|qb_{e-c}|)\sqrt{\frac{1}{\epsilon_0\hbar\omega_{sp}}\frac{\pi R^3}{2}} \tag{s72}$$

Having seen before the coupling strength between a free electron and a QE (Equation (s63)), we can extract the dipole moment that the free electron induces in the cavity:

$$|\boldsymbol{\mu}_c| = \frac{2\pi}{3}\sqrt{\pi R^3\,\hbar\omega_{sp}\epsilon_0} \tag{s73}$$

We further note that the amplitudes of this dipole moment satisfy: $|\boldsymbol{\mu}_c\cdot\hat{x}| = |\boldsymbol{\mu}_c\cdot\hat{z}|$ and $\boldsymbol{\mu}_c\cdot\hat{y} = 0$. From here one can see that for the parameters used in the main text $|\boldsymbol{\mu}_c^{ind}| \approx 40|\boldsymbol{\mu}_{QE}|$. This expression agrees well with the one derived from comparing the polarizability of a sphere with that of a quantum 2-level system [10].

If one calculates the integrated loss probability of an electron interacting with a spherical metallic nanoparticle in the non-retarded limit [6], and applies the good resonator approximation to the Drude description, the loss probability is calculated as:

$$P_L = \int d\omega\Gamma_{NR}^{sph}(\omega) = \int d\omega\frac{e^2}{\pi^2\epsilon_0\hbar v_0^2}\left[\left(\frac{\omega}{v}\right)^2 K_0^2\left(\frac{\omega b}{v}\right) + \left(\frac{\omega}{v}\right)^2 K_1^2\left(\frac{\omega b}{v}\right)\right]\mathrm{Im}\left\{\alpha_{NR}(\omega)\right\} \tag{s74}$$

$$\alpha_{NR}(\omega) = R^3\frac{\epsilon(\omega)-1}{\epsilon(\omega)+2} \approx i\frac{\omega_{sp}\pi R^3}{2}\delta(\omega-\omega_{sp}) \tag{s75}$$

$$P_L = \frac{e^2}{\pi^2\epsilon_0\hbar\omega_{sp}}\left(\frac{\omega_{sp}^4}{v^4}\right)\left[K_0^2\left(\frac{\omega_{sp}b}{v_0}\right) + K_1^2\left(\frac{\omega_{sp}b}{v_0}\right)\right]\frac{\pi R^3}{2} \tag{s76}$$

Looking at the electron-cavity couplings above, and taking the limit of small coupling, one can see that the first order of interaction between a free electron and a spherical nanoparticle, with the nanoparticle initially in it's ground state yields a probability for the electron to loose one photon that looks like

$$P_L \approx \left|\frac{L}{v_0}g_{\omega_{sp}/v_0,x}^{e-c}\right|^2 + \left|\frac{L}{v_0}g_{\omega_{sp}/v_0,z}^{e-c}\right|^2 \tag{s77}$$

$$= \frac{e^2}{9\epsilon_0\hbar\omega_{sp}}\left|\frac{\omega_{sp}}{v_0}\right|^4\left[K_0^2\left(\left|\frac{\omega_{sp}b_{e-c}}{v_0}\right|\right) + K_1^2\left(\left|\frac{\omega_{sp}b_{e-c}}{v_0}\right|\right)\right]\frac{\pi R^3}{2} \tag{s78}$$

Which agrees with the classical result to a factor of $(\pi/3)^2$. The agreement between our formalism and the classical result is not a coincidence, and besides the analytical similarity of the calculations involved in the derivation, what matters is that through MQED we have been able to define properly normalized bosonic modes, which means that once the electromagnetic Green's function of an arbitrary system is known, the interaction can be studied in the way that we have outlined.

S4.2.1 Intuitive picture of the coupling behavior

The effective dipole moment of the cavity in Eq. (s73) provides us with further understanding on the amplitude of the different intensity peaks in Fig. 3(b). For a target composed of a nanocavity and a QE initially in the ground state, the peaks in $I(\omega)$ are proportional (to leading order) to the electron couplings to the polaritonic modes that it supports. As we have shown in the main text, these can be written in

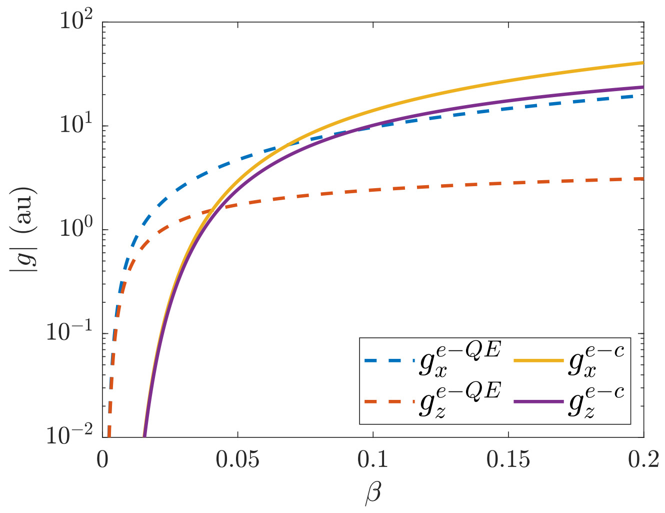

Fig. S2: Semiclassical coupling strength between the electron and the different entities in the target calculated per Equations (s79) to (s82) . Solid lines indicate the coupling to the cavity modes and dashed lines indicate the couplings to QEs oriented along the $\hat{x}$ and $\hat{z}$ directions. Note that in the main text the QE is oriented along $\hat{x}$.

terms of the bare electron couplings to the cavity modes and QE. Considering the free electron as a classical source, it is well known that one can characterize the light matter coupling as $g \propto \vec{\mu} \cdot \vec{E}$. Since the electric field created by a non-relativistic electron moving along the $\hat{z}$ direction is given by [6]:

$$\vec{E} = \frac{2e\omega e^{i\omega z/v}}{v^2}\left[iK_0\left(\frac{\omega b}{v}\right)\hat{z} - K_1\left(\frac{\omega b}{v}\right)\hat{R}\right],$$

then the different couplings are proportional to:

$$|g_x^{e-c}| \propto (\hat{\mu}_c \cdot \hat{x})\left|\frac{\mu_c}{\mu_{QE}}\right| K_1\left(\frac{k_0 b_{e-c}}{v/c}\right) \quad \text{(s79)}$$

$$|g_z^{e-c}| \propto (\hat{\mu}_c \cdot \hat{z})\left|\frac{\mu_c}{\mu_{QE}}\right| K_0\left(\frac{k_0 b_{e-c}}{v/c}\right) \quad \text{(s80)}$$

$$|g_x^{e-QE}| \propto (\hat{\mu}_{QE} \cdot \hat{x})\, K_1\left(\frac{k_0 b_{e-QE}}{v/c}\right) \quad \text{(s81)}$$

$$|g_z^{e-QE}| \propto (\hat{\mu}_{QE} \cdot \hat{z})\, K_0\left(\frac{k_0 b_{e-QE}}{v/c}\right) \quad \text{(s82)}$$

where $\hat{\mu}_c$ and $\hat{\mu}_{QE}$ are unitary vectors in the direction of the dipole moments. These expressions show that the electron couplings in our polaritonic target are dictated by three factors:

- The cavity dipole moment is significantly larger (more than one order of magnitude) that the QE dipole moment.
- The electric field generated by the travelling electron decays exponentially with distance from its trajectory, similarly for transverse and longitudinal field components.
- A larger electron velocity effectively reduces the optical path, $k_0 b$, between free electron and target.

In Figure S2 the coupling strengths above are evaluated for the parameters in the main text: $b_{e-c} = 11$ nm, $b_{e-QE} = 1$ nm, $\omega \approx 2$ eV, and $\mu_c/\mu_{QE} \approx 40$. It clarifies the main features in Fig. 3(b) on the main text:

- At low electron velocity, the dependence with the impact parameters dominates: In our setup we have $b_{e-QE} < b_{e-c}$, which vanishes the coupling to the optical modes. As a consequence, the electron populates the different polaritons only through the QE. This is why the two polaritons are equally bright at low electron velocity and the emission from the z-dipolar mode is strongly suppressed.

- As the electron velocity increases, the effective impact parameter diminishes, which exponentially increases the coupling strength. Since the dipole moment is much larger than that of the QE, the coupling strengths becoming comparable. Since the interaction with the polaritonic states in the first excitation manifold is given by the sum and difference of these coupling strengths, in this regime where the coupling to one of them becomes negligible. Note that this cross-over takes place at two different electron velocities depending on the QE orientation (x or z).
- For larger electron velocities, the coupling to the x-dipolar mode becomes dominant, but the coupling to the QE remains relevant. Therefore, the polaritonic states are populated asymmetrically.

S4.3 Validity of the quasi-static approximation

In this section, we explore the validity of the quasi-static approximation we have used for the Hamiltonian parametrization. The two conditions that need to be fulfilled in order to safely neglect retardation effects are $k_0 b \ll 1$, and $k_0 R \ll 1$. In our case $R = 10$ nm, and $b = 11$ nm, and, at the frequency range of our study ($\hbar\omega_{sp} = 2$ eV), these inequalities evaluate to $k_0 b \approx 0.1 > k_0 R$, which makes the quasi-static approximation valid. To further verify this conclusion, we borrow the analytical expression [6] describing the energy loss probability for a free electron in the vicinity of a spherical nanoparticle including retardation effects

$$\Gamma^{\mathrm{R}}_{\mathrm{sph}}(\omega) = \frac{e^2}{4\pi\epsilon_0}\frac{R}{\pi\hbar c^2}\frac{\pi}{k_0 R}\sum_{l=1}^{\infty}\sum_{m=-l}^{l} K_m^2\left(\frac{k_0 b}{\beta\gamma}\right)\left[C^M_{lm}\mathrm{Im}(t_l^M) + C^E_{lm}\mathrm{Im}(t_l^E)\right],$$

where the definition of the different terms can be found in Ref. [6]. In the limit of small radius, R, the magnetic scattering coefficients, t_l^M, vanish, and the the dominant contribution is given by the electric dipole mode, which acquires the form

$$t_1^E \overset{k_0 R\to 0}{\approx} \frac{2}{3}(k_0 R)^3\frac{\epsilon(\omega)-1}{\epsilon(\omega)+2},$$

where $\epsilon(\omega)$ is the nanoparticle permittivity. This way, the expression for the energy-loss probability in the quasi-static approximation can be obtained

$$\Gamma^{\mathrm{QS}}_{\mathrm{sph.dip}}(\omega) = \frac{e^2}{4\pi\epsilon_0}\frac{R}{\pi\hbar c^2}\left(\frac{k_0 R}{\beta}\right)^2\frac{4}{\beta^2}\left[K_1^2\left(\frac{k_0 b}{\beta}\right) + K_0^2\left(\frac{k_0 b}{\beta}\right)\right]\mathrm{Im}\left(\frac{\epsilon(\omega)-1}{\epsilon(\omega)+2}\right),$$

where $\beta = v/c$.

Employing a hydrodynamical Drude model for the metal permittivity [11], the key parameter measuring the impact of nonlocality is $\mathcal{B} = \sqrt{3/5}\, v_F$, which depends on the Fermi velocity of the metal. Using a 3D Fermi gas model, this can be expressed in terms of the electron density as $v_F = \hbar/m(3\pi^2 n)^{1/3}$. Thus, the characteristic length-scale for nonlocality (which sets the emergence of relevant electron-electron interactions in the metal response) can be estimated as [12]

$$l_F \sim \frac{\mathcal{B}}{\omega_p} = (3\pi^2)^{1/3}\frac{\hbar}{e}\sqrt{\frac{\epsilon_0}{m}}n^{-1/6}$$

where $\omega_p = \sqrt{ne^2/\epsilon_0 m}$ is the Drude plasma frequency. For noble metals, such as silver or gold, $\mathcal{B} \approx 0.01c$ and $l_F \approx 0.1$ nm, much smaller than R and b in our polaritonic model system.

One of the first theoretical approaches accounting for non-local effects in the optical response of metallic nanospheres was proposed by Ruppin [13]. Employing a hidrodynamical Drude model, scattering coefficients, t_l^E and t_l^M, were obtained that included a non-local length-scale correction of the form

$$\delta_l^{NL} = l(l+1)\left(\epsilon(\omega)-1\right)\frac{j_l(x_{nl})}{x_{nl}j_l'(x_{nl})}, \text{ with } x_{nl} = \sqrt{\frac{\epsilon(\omega)}{1-\epsilon(\omega)}\frac{\omega_p R}{\mathcal{B}}}.$$

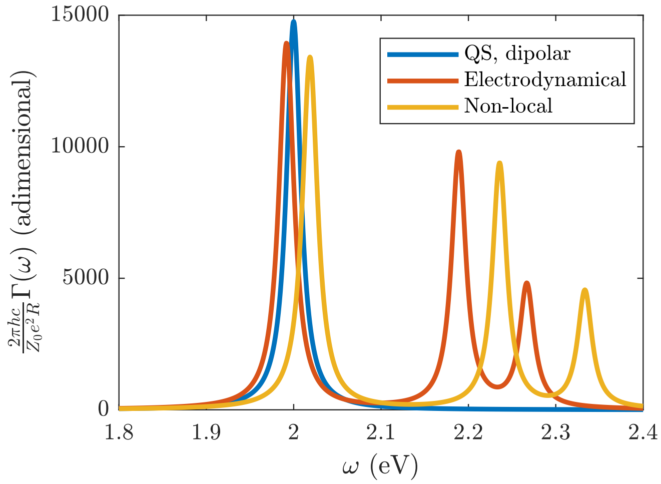

Fig. S3: Electron energy loss probability for an electron with $\beta = 0.1$ interacting with a sphere of radius $R = 10$ nm at an impact parameter $b = 11$ nm. The result is shown in the quasi-static limit, in the full electrodynamical picture, and with non-local corrections.

With the elements introduced above, we can compare electron energy loss probabilities for the nanophotonic cavity model in the main text, obtained under quasi-static, retarded, and non-local descriptions. We showcase the result up to the octupolar response in Figure S3. In agreement with our initial estimations, the electrodynamic, fully-retarded $\Gamma(\omega)$ does not deviate significantly from the quasi-static prediction. The only fingerprint of retardation is a few meV redshift in the dipolar mode (2 eV) of the cavity, which validates neglecting retardation effects in the calculation of the electron-target coupling strengths. Similarly, the comparison with the non-local-corrected model, reveals that nonlocality does not have a significant impact either at frequencies in the vicinity of the dipolar resonance. It yields a blueshift of the dipolar mode, comparable in magnitude, to the redshift induced by retardation. Thus, in the ~ 2 eV range, both effects tend to counterbalance each other. Note however, that the deviation between retarded and nonlocal predictions become larger at higher frequencies, at the quadrupolar and octupolar cavity modes, which are not considered in the target system in the main text.

S5 Scattering matrix of the interaction between a free electron and quantum systems

We start from the general expression of the scattering matrix given in the main text, and use the completitude relation by introducing a sum over the eigenstates of the $\hat{H}_0$ hamiltonian:

$$\hat{S}(t) = \mathcal{T}\exp\left(-\frac{i}{\hbar}\int_{-\infty}^{t} e^{i\hat{H}_0\tau/\hbar}\hat{H}_I e^{-i\hat{H}_0\tau/\hbar} d\tau\right) \tag{s83}$$

$$= \mathcal{T}\exp\left(-\frac{i}{\hbar}\sum_{\phi,\phi'}\int_{-\infty}^{t} |\phi'\rangle\langle\phi'| e^{i\hat{H}_0\tau/\hbar}\hat{H}_I e^{-i\hat{H}_0\tau/\hbar} |\phi\rangle\langle\phi| d\tau\right) \tag{s84}$$

$$= \mathcal{T}\exp\left(-\frac{i}{\hbar}\sum_{\phi,\phi'} |\phi'\rangle\langle\phi'| \hat{H}_I |\phi\rangle\langle\phi| \int_{-\infty}^{t} e^{-i\left(\frac{E_\phi - E_{\phi'}}{\hbar}\right)\tau} d\tau\right) \tag{s85}$$

where we have introduced E_ϕ to denote the energy of the eigenstate ϕ of the free hamiltonian $\hat{H}_0$. Taking the limit of $t \to \infty$ the above integral turns into a delta function imposing energy conservation:

$$\hat{S} \equiv \lim_{t\to\infty} \hat{S}(t) = \exp\left(-\frac{i}{\hbar}\sum_{\phi,\phi'} |\phi'\rangle\langle\phi'|\, \hat{H}_I\, |\phi\rangle\langle\phi|\, 2\pi\delta\left(\frac{E_\phi - E_{\phi'}}{\hbar}\right)\right) \tag{s86}$$

where time ordering becomes irrelevant as there is no time dependence on the total propagator. Since we use a fictitious box to normalize the electron's wavefunction, then the electron's momenta is discrete, and so is the energy spectra. As mentioned in the main text, this allows to make the substitution $\delta((E_\phi - E_{\phi'})/\hbar) \to (L/2\pi v_0)\delta_{E_\phi,E_{\phi'}}$, which leads to the scattering matrix present on the main text:

$$\hat{S} = \exp\Big(-i\sum_{\phi,\phi'} h_{I,\phi,\phi'}\, |\phi\rangle\langle\phi'|\Big), \tag{s87}$$

$$h_{I,\phi,\phi'} = \delta_{E_\phi,E_{\phi'}} \frac{L}{\hbar v_0} \langle\phi|\hat{H}_I|\phi'\rangle\,. \tag{s88}$$

S6 Algebraic structure of scattering matrix and general properties of electrons interacting with quantum systems

In the main text we show that the scattering matrix can be written as

$$\hat{S} = \exp\left(-i\hat{O}\right) \tag{s89}$$

$$\hat{O} = \frac{L}{\hbar v_0}\sum_{\phi,\phi'} \delta_{E_\phi,E_{\phi'}}\, |\phi\rangle\langle\phi|\, \hat{H}_I\, |\phi'\rangle\langle\phi'| \tag{s90}$$

with the interaction hamiltonian given by

$$\hat{H}_I = \hbar\sum_{i=x,z}\sum_q g^{c-e}_{q,i}\hat{b}_q\left[\hat{a}_i^\dagger - \hat{a}_i\right]\mathrm{sign}(q) - \hbar\sum_q g_q^{e-QE}\hat{b}_q\left\{\sigma - \sigma^\dagger\right\} = \tag{s91}$$

$$\equiv \sum_q \hat{H}_{I,q}\hat{b}_q \tag{s92}$$

where we have grouped all the operators that act on the cavity-QE system inside $\hat{H}_{I,q}$. We could then explicitly write out the $|\phi\rangle$ initial and final states in terms of the bare cavity ($|n_z\rangle$), polaritons ($|N,\pm\rangle$) and electronic momenta ($|k\rangle$) as $|n_z, N, \pm, k\rangle$, but for compactness, we choose to only explicitly write the electron state, and introduce $|\psi\rangle$ as a compact representation of the rest of the system. Then the $\hat{O}$ operator can be writen as:

$$\hat{O} = \frac{L}{\hbar v_0}\sum_{\substack{\psi,k\\ \psi',k'}} \delta_{E(\psi,k),E(\psi',k'))}\, |\psi,k\rangle\langle\psi,k|\, \hat{H}_I\, |\psi',k'\rangle\langle\psi',k'| \tag{s93}$$

$$= \frac{L}{\hbar v_0}\sum_{\substack{\psi,k\\ \psi',k'}}\sum_q \delta_{E(\psi,k),E(\psi',k'))}\, |\psi,k\rangle\,\langle\psi,k|\, \hat{H}_{I,q}\, |\psi',k'-q\rangle\,\langle\psi',k'| \tag{s94}$$

$$= \frac{L}{\hbar v_0}\sum_{\substack{\psi\\ \psi',k'}}\sum_q \delta_{E(\psi,k'-q),E(\psi',k'))}\, |\psi\rangle\langle\psi|\, \hat{H}_{I,q}\, |\psi'\rangle\langle\psi'| \otimes |k'-q\rangle\,\langle k'| \tag{s95}$$

Using the free electron energy dispersion, and the non-recoil approximation, one can see that energy conservation imposes

$$q \equiv q_{\psi,\psi'} = \frac{E(\psi) - E(\psi')}{\hbar v_0} \tag{s96}$$

with v_0 being the central velocity of the incident electron. This is independent of the initial momentum k', and therefore the evolution operator $\hat{O}$ can be cast as:

$$\hat{O} = \frac{L}{\hbar v_0} \sum_{\psi,\psi'} |\psi\rangle\langle\psi| \hat{H}_{I,q_{\psi,\psi'}} \otimes \hat{b}_{q_{\psi,\psi'}} |\psi'\rangle\langle\psi'| \equiv \sum_{\psi,\psi'} \hbar_{I,\psi,\psi'} |\psi\rangle\langle\psi'| \otimes \hat{b}_{q_{\psi,\psi'}} \tag{s97}$$

$$\hbar_{I,\psi,\psi'} = \frac{L}{\hbar v_0} \langle\psi| \hat{H}_{I,q_{\psi,\psi'}} |\psi'\rangle \tag{s98}$$

Which shows that through energy conservation, each possible transition within the reduced system is accompanied by a corresponding shift in the electron. This comes from the particular algebra of the electron's ladder operators and the non-recoil approximation alone, and therefore the expression above applies to electrons interacting with any particular kind of quantum system. Since the scattering matrix describing the final state of the system after the interaction of the electron with a QS is given by the exponentiation of the $\hat{O}$ operator, and we expect small interaction strength between electron-QS, it makes sense to take a look at the behavior of powers of $\hat{O}$. If we denote the basis of eigenstates of the quantum system by $|\psi_i\rangle$, then some powers can be written as:

$$\hat{O} = \sum_{\psi_i,\psi_j} h_{I,\psi_i,\psi_j} |\psi_i\rangle\langle\psi_j| \otimes \hat{b}_{q_{\psi_i,\psi_j}} \tag{s99}$$

$$\hat{O}^2 = \sum_{\psi_i,\psi_j} \left[\sum_{\psi_k} h_{I,\psi_i,\psi_k} h_{I,\psi_k,\psi_j} \right] |\psi_i\rangle\langle\psi_j| \otimes \hat{b}_{q_{\psi_i,\psi_j}} \tag{s100}$$

$$\hat{O}^3 = \sum_{\psi_i,\psi_j} \left[\sum_{\psi_k,\psi_l} h_{I,\psi_i,\psi_k} h_{I,\psi_k,\psi_l} h_{I,\psi_l,\psi_j} \right] |\psi_i\rangle\langle\psi_j| \otimes \hat{b}_{q_{\psi_i,\psi_j}} \tag{s101}$$

$$\hat{O}^4 = \sum_{\psi_i,\psi_j} \left[\sum_{\psi_k,\psi_l,\psi_m} h_{I,\psi_i,\psi_k} h_{I,\psi_k,\psi_l} h_{I,\psi_l,\psi_m} h_{I,\psi_m,\psi_j} \right] |\psi_i\rangle\langle\psi_j| \otimes \hat{b}_{q_{\psi_i,\psi_j}} \tag{s102}$$

Notation becomes cumbersome, but the main idea is that regardless of how many photon exchanges happen between the electron and QS (the power of operator $\hat{O}$ above), the commutativity of the electron ladder operators guarantee that the final shift that the electron experiences will only depend on the energy difference between the initial and final states connected by the interaction, and therefore $\hat{O}^n$ will always have the form,

$$\hat{O}^n = \sum_{\psi_i,\psi_j} h^{(n)}_{I,\psi_i,\psi_j} |\psi_i\rangle\langle\psi_j| \otimes \hat{b}_{q_{\psi_i,\psi_j}} \tag{s103}$$

And therefore, the general form of the propagator becomes:

$$\hat{S} = \exp\left(-\frac{i}{\hbar}\frac{L}{v}\hat{O}\right) = \sum_{\psi_i,\psi_j} \left[\sum_{n=0}^{\infty} \left(-\frac{i}{\hbar}\frac{L}{v}\right)^n \frac{h^{(n)}_{I,\psi_i,\psi_j}}{n!} \right] |\psi_i\rangle\langle\psi_j| \otimes \hat{b}_{q_{\psi_i,\psi_j}} \equiv \tag{s104}$$

$$\equiv \sum_{\psi_i,\psi_j} S_{\psi_i,\psi_j} |\psi_i\rangle\langle\psi_j| \otimes \hat{b}_{q_{\psi_i,\psi_j}} \tag{s105}$$

This structure has direct implications on how the interaction with the electron might create or modify populations on any QS. Suppose for instance that initially the QS is prepared on one of the basis states, $|\psi_n\rangle$, and the electron is prepared with an arbitrary momentum distribution $|\psi_e\rangle = \int dk B(k) |k\rangle$. The final state

of the system after interaction will be given by $|\psi\rangle = \sum_{\psi_i} S_{\psi_i,\psi_n} \hat{b}_{q_{\psi_i,\psi_n}} |\psi_i, \psi_e\rangle$, and the corresponding density matrix, and reduced density matrix for the QS are given by:

$$\rho = |\psi\rangle\langle\psi| = \sum_{\psi_i,\psi_j} S_{\psi_i,\psi_n} S^*_{\psi_j,\psi_n} \hat{b}_{q_{\psi_i,\psi_n}} |\psi_i, \psi_e\rangle\langle\psi_j, \psi_e| \hat{b}^\dagger_{q_{\psi_j,\psi_n}} \tag{s106}$$

$$\rho^{QS} = \sum_{\psi_i,\psi_j} S_{\psi_i,\psi_n} S^*_{\psi_j,\psi_n} |\psi_i\rangle\langle\psi_j| \int dk B(k) B^*(k - [q_{\psi_i,\psi_n} - q_{\psi_j,\psi_n}]) \tag{s107}$$

The second expression above, despite the absolute agnosticity about the particular QS the electron interacts with, allows to assert that if the QS is initially in only one of it's eigenstates, then the final populations are absolutely independent of the particular electronic wavefunction and only the coherences will be sensitive to the initially prepared electron. Now instead consider that the QS is initially prepared in a superposition of two of it's basis states: $|\psi_{n_1}\rangle$ and $|\psi_{n_2}\rangle$ as $\left|\psi_0^{QS}\right\rangle = \cos(\theta)|\psi_{n_1}\rangle + \sin(\theta)e^{i\phi}|\psi_{n_2}\rangle$. Then the final state $|\psi\rangle$, density matrix ρ and reduced density matrix ρ^{QS} read:

$$|\psi\rangle = \sum_{\psi_i} \left[\cos(\theta) S_{\psi_i,\psi_{n_1}} \hat{b}_{q_{\psi_i,\psi_{n_1}}} + \sin(\theta) e^{i\phi} S_{\psi_i,\psi_{n_2}} \hat{b}_{q_{\psi_i,\psi_{n_2}}}\right] |\psi_i, \psi_e\rangle \tag{s108}$$

$$\begin{aligned}
\rho = \sum_{\psi_i,\psi_j} \Big\{ & \cos^2(\theta) S_{\psi_i,\psi_{n_1}} S^*_{\psi_j,\psi_{n_1}} \hat{b}_{q_{\psi_i,\psi_{n_1}}} |\psi_i, \psi_e\rangle\langle\psi_j, \psi_e| \hat{b}^\dagger_{q_{\psi_j,\psi_{n_1}}} \\
& + \sin^2(\theta) S_{\psi_i,\psi_{n_2}} S^*_{\psi_j,\psi_{n_2}} \hat{b}_{q_{\psi_i,\psi_{n_2}}} |\psi_i, \psi_e\rangle\langle\psi_j, \psi_e| \hat{b}^\dagger_{q_{\psi_j,\psi_{n_2}}} \\
& + \cos(\theta)\sin(\theta) e^{-i\phi} S_{\psi_i,\psi_{n_1}} S^*_{\psi_j,\psi_{n_2}} \hat{b}_{q_{\psi_i,\psi_{n_1}}} |\psi_i, \psi_e\rangle\langle\psi_j, \psi_e| \hat{b}^\dagger_{q_{\psi_j,\psi_{n_2}}} \\
& + \cos(\theta)\sin(\theta) e^{i\phi} S_{\psi_i,\psi_{n_2}} S^*_{\psi_j,\psi_{n_1}} \hat{b}_{q_{\psi_i,\psi_{n_2}}} |\psi_i, \psi_e\rangle\langle\psi_j, \psi_e| \hat{b}^\dagger_{q_{\psi_j,\psi_{n_1}}} \Big\}
\end{aligned} \tag{s109}$$

$$\begin{aligned}
\rho^{QS} = \sum_{\psi_i,\psi_j} \Big\{ & \cos^2(\theta) S_{\psi_i,\psi_{n_1}} S^*_{\psi_j,\psi_{n_1}} \int dk B(k) B^*(k - [q_{\psi_i,\psi_{n_1}} - q_{\psi_j,\psi_{n_1}}]) \\
& + \sin^2(\theta) S_{\psi_i,\psi_{n_2}} S^*_{\psi_j,\psi_{n_2}} \int dk B(k) B^*(k - [q_{\psi_i,\psi_{n_2}} - q_{\psi_j,\psi_{n_2}}]) \\
& + \cos(\theta)\sin(\theta) e^{-i\phi} S_{\psi_i,\psi_{n_1}} S^*_{\psi_j,\psi_{n_2}} \int dk B(k) B^*(k - [q_{\psi_i,\psi_{n_1}} - q_{\psi_j,\psi_{n_2}}]) \\
& + \cos(\theta)\sin(\theta) e^{i\phi} S_{\psi_i,\psi_{n_2}} S^*_{\psi_j,\psi_{n_1}} \int dk B(k) B^*(k - [q_{\psi_i,\psi_{n_2}} - q_{\psi_j,\psi_{n_1}}]) \Big\} |\psi_i\rangle\langle\psi_j|
\end{aligned} \tag{s110}$$

Particularly, the populations are given by:

$$\begin{aligned}
\langle\psi_i| \rho^{QS} |\psi_i\rangle = & \cos^2(\theta) S_{\psi_i,\psi_{n_1}} S^*_{\psi_i,\psi_{n_1}} + \sin^2(\theta) S_{\psi_i,\psi_{n_2}} S^*_{\psi_i,\psi_{n_2}} \\
& + \cos(\theta)\sin(\theta) e^{-i\phi} S_{\psi_i,\psi_{n_1}} S^*_{\psi_i,\psi_{n_2}} \int dk B(k) B^*(k - q_{\psi_{n_2},\psi_{n_1}}) \\
& + \cos(\theta)\sin(\theta) e^{i\phi} S_{\psi_i,\psi_{n_2}} S^*_{\psi_i,\psi_{n_1}} \int dk B^*(k) B(k - q_{\psi_{n_2},\psi_{n_1}}) \qquad \text{(s111)} \\
= & \cos^2(\theta) \left|S_{\psi_i,\psi_{n_1}}\right|^2 + \sin^2(\theta) \left|S_{\psi_i,\psi_{n_2}}\right|^2 \\
& + \sin(2\theta) \operatorname{Re}\left\{ e^{-i\phi} S_{\psi_i,\psi_{n_1}} S^*_{\psi_i,\psi_{n_2}} \int dk B(k) B^*(k - q_{\psi_{n_2},\psi_{n_1}}) \right\} \qquad \text{(s112)}
\end{aligned}$$

which explicitly shows that for arbitrary quantum systems, the electronic wavefunctions will give contributions to the final populations of the reduced system so long as the initial QS is found in a superposition of states, and the modulation of the electron is tuned appropriately to the energy difference between populated states.

S7 Computational implementation of the calculations

The main advantage of the present work as compared to recent contributions to the study of polaritons through the use of electrons is that we have access to analytical expressions of the different quantities. Their particular expressions are too large to result useful to the general reader, but the way in which the calculation is implemented does have it's advantages. The main idea is to exploit two basic properties of the system:

- The initial state of the system can always be characterized as a product state of the electron and the rest of the system.
- The algebraic properties of the electronic ladder operators allows to treat them in a particularly useful way.

Since the initial state of a free electron interacting with a quantum system (QS) can always be described as a product state between the electron and the QS, one can perform all the algebraic manipulations on the QS's Hilbert space and then apply the resulting $\hat{b}_q$ operators over the electronic wavefunction. In systems with only one energy scale, such as isolated quantum emitters or single mode fields, this strategy doesn't really save much work since the free electron's state can be characterized by just how many photons it has exchanged with the QS. However, when dealing with QS with several energy scales, then the electron can exchange photons of several different energies (which also depend on the QS's initial state), and keeping track of all the possible final energies of the electron becomes a rather difficult task. Furthermore, the size of the electron's Hilbert space will also depend on the initial electron's momentum distribution. All this indicates that trying to define the electron's Hilbert space beforehand will lead to problems of gargantuan proportions, since in principle one has to account for a continuum of possible final electron momenta. This is where the strategy outlined above shines: By performing all the algebra on the reduced Hilbert space of the QS (In out case all the states up to the 4th excitation manifold of the cavity + QE), one can obtain all the possible processes taking place within the QS, and find the associated effect these have on an arbitrary electron wavefunction. This allows us to effectively tackle the infinite-dimensional electron's Hilbert space for arbitrary initial states for the electron and QS and get analytical expressions for the different observables.

In order to computationally implement this strategy, we exploited the structure of Equation (s105) and the symbolic engine of Matlab. From Equation (s105) we see that the general scattering matrix can be obtained by first writing the interaction hamiltonian in matrix form in a basis of the reduced quantum system, and then by exponentiating this matrix one obtains the different matrix elements of the Scattering matrix. To complete the scattering matrix one needs to multiply each entry of this matrix by the appropriate electron ladder operator dictated by energy conservation. To perform the last part, we defined a new class of objects, "b_{obj}". These objects inherit the algebra of the $\hat{b}_q$ operators and allow to describe the interaction of the electron with a general system, without much thought about the particular electronic wavefunction or different momentum exchanges that might take place. We now give an outline of how these are implemented. These objecs are defined by two kinds of parameters: the amplitude, α, and momentum exchange, q_0, so that:

$$\alpha\hat{b}_{q_0} \equiv b_{obj}(\alpha, q_0)$$

where α is any complex number describing the amplitude of the operator and q_0 is the momentum shift of the ladder operator. These objects follow the following properties:

1. Hermitian conjugation: $(\alpha\hat{b}_q)^\dagger = \alpha^*\hat{b}_q^\dagger = \alpha^*\hat{b}_{-q} \to \left(b_{obj}(\alpha, q_0)\right)^\dagger \equiv b_{obj}(\alpha^*, -q_0)$.
2. Commutativity of ladder operators: $(\alpha\hat{b}_{q_0})(\beta\hat{b}_{q_1}) = \alpha\beta\hat{b}_{q_0+q_1} \to b_{obj}(\alpha, q_0)b_{obj}(\beta, q_1) \equiv b_{obj}(\alpha\beta, q_0 + q_1)$.
3. Addition of several ladder operators: $b_{obj}(\alpha, q_0) + b_{obj}(\beta, q_1) \equiv b_{obj}([\alpha, \beta], [q_0, q_1])$.
4. Distributive property: $b_{obj}(\gamma, q_2)b_{obj}([\alpha, \beta], [q_0, q_1]) \equiv b_{obj}([\alpha\gamma, \gamma\beta], [q_0 + q_2, q_1 + q_2])$.

With these rules and performing the algebra on the reduced QS, in the end we have one of these objects with a list of the different momentum exchanges taking place in the system, and a list of the amplitudes corresponding to them.

References

[1] H. T. Dung, L. Knöll, and D. G. Welsch, Physical Review A - Atomic, Molecular, and Optical Physics **66**, 16 (2002).
[2] J. Feist, A. I. Fernández-Domínguez, and F. J. García-Vidal, Nanophotonics **10**, 477 (2020).
[3] S. Y. Buhmann, *Dispersion Forces I*, Vol. 247 (Springer Berlin Heidelberg, 2012).
[4] L. Novotny and B. Hecht, *Principles of nano-optics* (2009).
[5] S. Y. Buhmann and D.-G. Welsch, Physical Review A **77**, 012110 (2008).
[6] F. J. García de Abajo, Rev. Mod. Phys. **82**, 209 (2010).
[7] O. Kfir, H. Lourenço-Martins, G. Storeck, M. Sivis, T. R. Harvey, T. J. Kippenberg, A. Feist, and C. Ropers, Nature **582**, 46 (2020).
[8] M. Abramowitz, I. A. Stegun, and R. H. Romer, American Journal of Physics **56**, 958 (1988).
[9] D. Zhao, R. E. F. Silva, C. Climent, J. Feist, A. I. Fernández-Domínguez, and F. J. García-Vidal, ACS Photonics **7**, 3369 (2020), pMID: 33365360, https://doi.org/10.1021/acsphotonics.0c01095 .
[10] C. Gonzalez-Ballestero, J. Feist, E. Moreno, and F. J. Garcia-Vidal, Phys. Rev. B **92**, 121402 (2015).
[11] C. Ciraci, R. Hill, J. Mock, Y. Urzhumov, A. Fernandez-Dominguez, S. Maier, J. Pendry, A. Chilkoti, and D. Smith, SCIENCE **337**, 1072 (2012).
[12] A. I. Fernández-Domínguez, A. Wiener, F. J. García-Vidal, S. A. Maier, and J. B. Pendry, Phys. Rev. Lett. **108**, 106802 (2012).
[13] R. Ruppin, Phys. Rev. Lett. **31**, 1434 (1973).
